# Does insulin-like growth factor moderate the association between height and risk of cancer at 24 sites?

**DOI:** 10.1038/s41416-020-01059-1

**Published:** 2020-09-14

**Authors:** Solange Parra-Soto, Frederick K. Ho, Jill P. Pell, Carlos Celis-Morales

**Affiliations:** 1 Institute of Health and Wellbeing, University of Glasgow, Glasgow, G12 8RZ UK; 2 Institute of Cardiovascular and Medical Sciences, University of Glasgow, Glasgow, G12 8TA UK; 3 Centre of Exercise Physiology Research (CIFE), Universidad Mayor, Santiago, Chile; 4 Laboratorio de Rendimiento Humano, Grupo de Estudio en Educación, Actividad Física y Salud (GEEAFyS), Universidad Católica del Maule, Talca, Chile

**Keywords:** Risk factors, Predictive markers

## Abstract

**Background:**

Whether the association of height with cancers differs by insulin-like growth factors has not been fully elucidated. Therefore, this study aimed to investigate the sex-specific associations between height and 24 site-specific cancers and to assess whether the association differed by IGF-1.

**Methods:**

In total, 414,923 participants from the UK Biobank prospective cohort study were included. The association of height (per 5-cm increment) with incidence and mortality from 24 cancer sites was investigated by using Cox proportional hazard models.

**Results:**

The median follow-up was 6.0 years. In men, height was positively associated with incidence risk of all-cause cancer and at five sites (lung, lymphatic, leukaemia, non-Hodgkin lymphoma and melanoma). In women, it was associated with breast, melanoma, lymphatic, non-Hodgkin lymphoma and all-cause cancer. The association was stronger in women than men for all-cause cancer incidence. The strength of the association did not differ by IGF-1 concentration.

**Conclusions:**

Adult height was associated with risk of several cancer sites. However, some of these associations were sex-specific. There was no strong evidence to support IGF-1 moderating the association between height and cancer.

## Background

Although simple to measure, height is a complex phenotype that is downstream of multiple biological and sociological determinants.^1^ Height has been associated with many chronic diseases, including cardiovascular diseases and cancer. Although height has a strong genetic component;^2,3^ the environment, especially economic development and nutrition, plays a major role in determining our height.^1^ Recent evidence suggests that changes in a population’s height also correlate with cancer estimates.^4^

Height has been robustly associated with a higher risk for many cancer sites, with most of this evidence derived from prospective cohort studies,^2,5,6^ but also recently from Mendelian Randomization (MR) studies.^2,3,7^ The last report from the World Cancer Research Fund (WCRF) showed that eight cancer sites are positively associated with height (colorectum, breast, ovary, pancreas, endometrium, prostate, kidney and skin). However, five of these cancer sites have been labelled as having ‘probable evidence'; therefore, further research is required.^6^ A recent study, published for Choi et al., investigated the association between height and cancer risk in a cohort of 23 million Korean adults.^8^ This study reported an increased risk of cancers of the nervous system, thyroid, breast, lung, colon, rectum, prostate, ovary, testes, cervix, endometrium, skin, lymphoma, multiple myeloma and leukaemia.^8^

However, the mechanism underpinning how height confers a higher risk of cancer is complex because the biological determinants of height are multifactorial. Despite this, several hypotheses have been proposed: one of them relates to insulin-like growth factor (IGF-1), which has a direct effect on increasing not only cancer risk but also height.^9^ IGF-1 is one of the most important determinants of height and organ size, and it has been postulated as a potential moderator of the link between height and cancer risk.^10,11^ A second hypothesis suggests that the increased risk conferred by height is attributable to more cells in taller, compared with shorter, people.^12–14^ Taller individuals have more stem cells and, therefore, they are exposed to a higher number of cell divisions during which driver mutations may occur.^11,13,14^ Moreover, the higher IGF-1 concentration and higher number of cells in taller people could, at least partly, explain differences in the cancer risk observed between men and women. However, whether IGF-1 modifies the association between height and cancer risk has not been fully elucidated.^11^ Therefore, by using the UK Biobank prospective cohort study, we aim to investigate the sex-specific association between height and 24 site-specific cancers to assess whether the association differed by circulating concentrations of IGF-1.

## Methods

### Data sources

In total, 502,536 participants (aged 37–73 years, 56.3% were women) were recruited into the UK Biobank between 2006 and 2010. Participants attended one of 22 assessment centres across England, Scotland and Wales, where they completed a touch-screen questionnaire, had physical measurements taken and provided biological samples, as described in detail elsewhere.^15,16^ The outcomes in the study reported here were incidence of and mortality from 24 site-specific cancers, with the exposure variable being height (expressed in 5-cm increment). We treated sociodemographic factors (age, ethnicity and area-based socioeconomic status), smoking status, waist circumference, self-reported physical activity, sedentary time, sleep and dietary intake as potential confounders, as well as prevalent comorbidities at baseline (diabetes, hypertension, cardiovascular diseases and long-standing illness). After excluding participants with prevalent cancer at baseline (*n* = 41,437) and those with missing data on covariates, exposures or outcomes (*n* = 46,176), 414,923 (82.6%) participants with full data available were included in this study.

### Procedures

The outcomes for this study were cancer incidence and mortality overall and for 24 site-specific cancers. Date and cause of death was obtained from death certificates held within the National Health Service Information Centre (England and Wales) and the National Health Service Central Register Scotland (Scotland). Dates and cause of hospital admissions were obtained from the Health Episode Statistics (England and Wales) and Scottish Morbidity Records (Scotland). Detailed information about the record linkage procedures can be found at http://content.digital.nhs.uk/services. Incident cancer was defined as the first record of the cancer of interest, from hospitalisation or death records. At the time of analysis, mortality data were available up to 14 February 2018. Mortality analyses were, therefore, censored at this date or date of death, whichever occurred earlier. Hospital admission data were available until 31 March 2017. Therefore, analyses of incident cancer were censored at this date, or the date of first hospitalisation for the cancer of interest or death, whichever occurred earlier.

The International Classification of Diseases, 10th revision (ICD-10), was used to define the following 24 cancer-specific sites: all cancers (C00–C97, D37, D48), brain (C71), oral (C00–C14), oesophagus (C15), stomach (C16), liver (C22), gallbladder (C23), pancreas (C25), lung (C34), colorectal (C18, C19 and C20), kidney (C64–C65), bladder (C67), thyroid (C73), lymphatic and haematopoietic tissue (C81–C96), non-Hodgkin lymphoma (C82–C85), multiple myeloma (C90), malignant melanoma (C43), leukaemia (C91–C95), prostate (C61), testis (C62), breast (C50), ovary (C56), endometrium (C54), uterine (C55) and cervix (C53). Of these, 20 cancer sites were used for men and women, two sites were specific to men (testis and prostate) and five to women (breast, endometrium, uterine, cervix and ovary).

### Exposure

Height was measured at baseline by trained staff using standardised protocols and a Seca 202 device (Seca, Hamburg, Germany).

### Covariables

Potential confounders were identified a priori based on established relationships, with cancer and height (Supplementary Fig. 1). Age, ethnicity, smoking status (non-smokers, ex-smokers and current smokers), dietary intake of major food groups, alcohol intake and female-specific factors were self-reported at the baseline assessment via a touch-screen questionnaire. Comorbidities and past medical history were based on self-report of physician diagnosis and verified during the face-to-face interview. Townsend area deprivation index was derived from the postcode of residence using aggregated data on unemployment, car and homeownership and household overcrowding, and was categorised into tertiles (low, middle and high).^17^ Physical activity level over a typical week was self-reported using the International Physical Activity Questionnaire and analysed as metabolic equivalent of task (MET) per week.^18^ Sedentary behaviour included time spent watching TV or in front of a PC at leisure time. Sleep time was also self-reported and categorised in short (<7 h/day), normal (7–9 h/day) and long sleepers (>9 h/day). Waist circumference was measured by trained nurses using a standard protocol. In the initial assessment visit (2006–2010),  over 500,000 participants were recruited and consented. Serum concentrations of insulin-like growth factor-1 (IGF-1) were measured using a DiaSorin Ltd (Beckman Coulter DXI 800) chemiluminescent immunoassay. The IGF-1 assays were externally validated with good correlation, and coefficients of variation were consistent across samples.^19^ Further details of these measurements can be found in the UK Biobank online protocol (http://www.ukbiobank.ac.uk).

### Statistical analyses

Cox proportional hazard models with follow-up time as the time-dependent variable were used to investigate sex-specific associations of height with incidence and mortality for 24 cancer sites and all-cause cancer. All analyses excluded participants who reported prevalent cancer at baseline. To minimise the potential contribution of reverse causality to the findings, we conducted a landmark analysis excluding people who had events within 2 years after recruitment.

Descriptive variables are presented as mean and standard deviation for continuous variables and number and percentage of participants for categorical variables. Pearson correlation was performed to investigate the associations of IGF-1 with age and height by sex. To investigate whether the concentration of IGF-1 by age and height differed by sex, interaction terms (age*sex and height*sex) were included in linear regression models.

Sex-specific associations between height (expressed per 5-cm increment) and cancer outcomes were studied using Cox proportional hazard models for both females and males independently. The results were reported as hazard ratios (HRs) and their 95% CI. All analyses were incrementally adjusted for the following covariates: Model 1 included age, ethnicity, deprivation and comorbidity (including prevalent hypertension, cardiovascular diseases, diabetes and long-standing illness); Model 2 included model 1 plus smoking, alcohol consumption, fruit and vegetables, processed meat, oily fish intake, sleep, physical activity and sedentary behaviours; Model 3 (fully adjusted) included variables from model 2 plus waist circumference.

To investigate differences on cancer risk between sex, men-to-women hazard ratios were then estimated using Cox models with height*sex interaction terms. This term represents the statistical interaction between sex and the predictor, and can be interpreted as the ratio of HR in men to  HR in women.

To investigate whether the association between height and cancer differed by IGF-1, we fitted an interaction term between height (per 5 cm) and age- and sex-standardised IGF-1 concentration. We also investigated whether the association between 5-cm increment in height and cancer differed by height (shorter or taller) by stratifying the analyses using the sex-specific median of height as the cut-off: above or below 162 cm for women and 176 cm for men. We also conducted sensitivity analyses for lung and women-specific cancers. The association of height with lung cancers was stratified by smoking status (current, ex-smoker and non-smoker). For women, the association between height and cervix, ovary, uterus, breast and endometrium cancers was stratified by pre- and postmenopausal status. In addition, stomach and liver cancers were stratified by reported alcohol consumption (≤1 per week vs. >1 per week). Finally, because of potentially inflated type-I errors due to multiple tests, all analyses were corrected for multiple testing using Holm’s method,^20^ which performed similarly as Bonferroni’s method while retaining higher statistical power.^21^ The multiple-testing-corrected *p* value is denoted as *P*_adj_.

All analyses were performed using R Statistical Software version 3.6.2 with the package survival. The proportional hazard assumption was verified by tests based on Schoenfeld residuals.

## Results

### Characteristics of the study population

Of the 502,536 participants enrolled in the UK Biobank, 414,923 were included in the current study. The median follow-up period was 6.03 (range 5.3–6.7) years for cancer incidence and 6.9 (range 6.3–7.5) years for cancer mortality. Over the follow-up period, 22,647 participants developed cancer and 4,539 died from it.

Baseline characteristics at baseline were described by sex in Table 1. The mean age was 56.3 years,  53.6% were women and 94.8% were of White-European ethnic background. The mean of height was 1.69 cm (1.76 cm and 1.62 cm for men and women, respectively). The mean concentrations of IGF-1 were 21.1 and 21.9 nmol/L for women and men, respectively. The Pearson correlation coefficients between IGF-1 and height were *r* = 0.118 and *r* = 0.107 for men and women, respectively (Supplementary Table S1). The associations of IGF-1 with age and height by sex are shown in Supplementary Figs S2, S3. In summary, IGF-1 concentration decreased in a linear manner with age for both men and women. However, the reduction in IGF was higher in women than men with increasing age (*p* interaction < 0.0001) (Supplementary Fig. S2). In contrast, IGF-1 concentration increased in a linear fashion with increasing height, with no differences between men and women (*p* interaction = 0.114) (Supplementary Fig. S3).

**Table 1:** Characteristics of participants.

	Females	Males	Overall
*n* (%)	222,635 (53.6%)	192,288 (46.3%)	414,923
Age (years), mean (SD)	56.1 (8.0)	56.5 (8.2)	56.3 (8.1)
IGF-1 (nmol/L), mean (SD)	21.1 (5.8)	21.9 (5.5)	21.5 (5.7)
Townsend deprivation index, *n* (%)			
Lower	75,311 (33.8%)	65,557 (34.1%)	140,868 (34.0%)
Middle	75,622 (34.0%)	63,654 (33.1%)	139,276 (33.6%)
Higher	71,702 (32.2%)	63,077 (32.8%)	134,779 (32.5%)
Ethnicity, *n* (%)			
White	211,041 (94.8%)	182,334 (94.8%)	393,375 (94.8%)
Mixed	3,532 (1.6%)	2,529 (1.3%)	6,061 (1.5%)
South Asian	3,645 (1.6%)	4,309 (2.2%)	7,954 (1.9%)
Black	3,613 (1.6%)	2,622 (1.4%)	6,235 (1.5%)
Chinese	804 (0.4%)	494 (0.3%)	1,298 (0.3%)
Height (m), mean (SD)	1.62 (0.1)	1.76 (0.1)	1.69 (0.1)
Weight (kg), mean (SD)	71.3 (14.0)	85.9 (14.2)	78.1 (15.9)
Waist (cm), mean (SD)	84.5 (12.4)	96.8 (11.2)	90.2 (13.4)
Body mass index (kg/m^2^), mean (SD)	27.0 (5.1)	27.8 (4.2)	27.4 (4.7)
Nutritional status, *n* (%)			
Underweight	1,629 (0.7%)	427 (0.2%)	2,056 (0.5%)
Normal	87,856 (39.5%)	48,179 (25.1%)	136,035 (32.8%)
Overweight	81,755 (36.7%)	95,382 (49.6%)	177,137 (42.7%)
Obese	51,395 (23.1%)	48,300 (25.1%)	99,695 (24.0%)
Smoking, *n* (%)			
Never	133,852 (60.1%)	95,290 (49.6%)	229,142 (55.2%)
Ex-smoker	69,315 (31.1%)	73,403 (38.2%)	142,718 (34.4%)
Current	19,468 (8.7%)	23,595 (12.3%)	43,063 (10.4%)
Alcohol intake, *n* (%)			
Daily or almost daily	36,136 (16.2%)	49,101 (25.5%)	85,237 (20.5%)
3–4 times a week	46,471 (20.9%)	51,095 (26.6%)	97,566 (23.5%)
Once or twice a week	57,861 (26.0%)	49,894 (25.9%)	107,755 (26.0%)
1–3 times a month	29,211 (13.1%)	17,053 (8.9%)	46,264 (11.2%)
Special occasions only	32,745 (14.7%)	13,614 (7.1%)	46,359 (11.2%)
Never	20,211 (9.1%)	11,531 (6.0%)	31,742 (7.7%)
Sleep time, *n* (%)			
Normal 7–9 h/day	165,418 (74.3%)	140,733 (73.2%)	306,151 (73.8%)
Short sleep <7 h/day	53,339 (24.0%)	48,480 (25.2%)	101,819 (24.5%)
Long sleep >9 h/day	3878 (1.7%)	3075 (1.6%)	6953 (1.7%)
Sedentary time (h/day), mean (SD)	4.66 (2.0)	5.46 (2.5)	5.03 (2.3)
Physical activity (MET h/week) mean (SD)	1.78 (1.7)	1.86 (1.6)	1.82 (1.7)
Diabetes, *n* (%)	7185 (3.2%)	12,823 (6.7%)	20,008 (4.8%)
Hypertension, *n* (%)	50,661 (22.8%)	57,155 (29.7%)	107,816 (26.0%)
CVD, *n* (%)	55,743 (25.0%)	64,937 (33.8%)	120,680 (29.1%)
Long-standing illness, *n* (%)	156,193 (70.2%)	124,823 (64.9%)	281,016 (67.7%)

Data are presented as the number of participants and their percentage (%) for categorical variables. Continuous variables are presented as mean and standard deviation. Data available for 414,923.

### Height and cancer risk

**Fig. 1:**
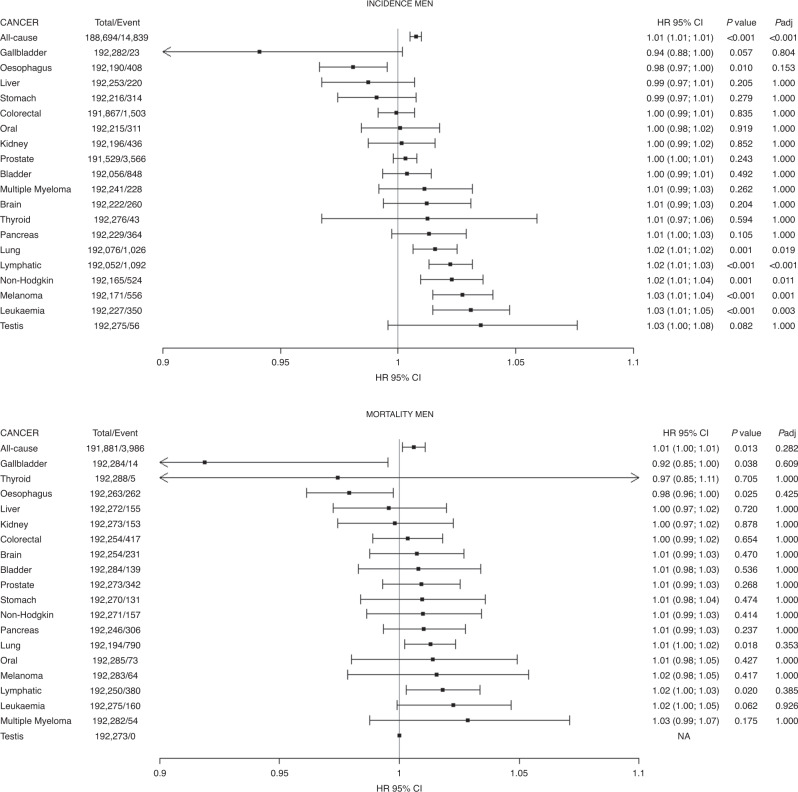
Association of height with the risk of incidence and mortality from 20 cancer sites in men. Data presented as hazard ratio and their 95% CI per 5-cm increment in height. Analyses were adjusted for age, sex, ethnicity, deprivation index, comorbidity, smoking, alcohol consumption, fruit and vegetable, processed meat intake, oily fish, sleep, physical activity, sedentary behaviours and waist circumference.

**Fig. 2:**
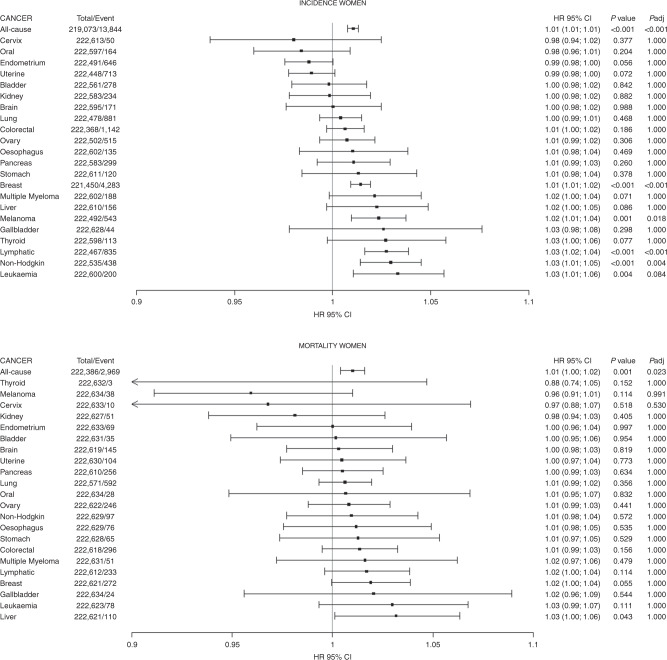
Association of height with the risk of incidence and mortality from 22 cancer sites in women. Data presented as hazard ratio and their 95% CI per 5-cm increment in height. Analyses were adjusted for age, sex, ethnicity, deprivation index, comorbidity, smoking, alcohol consumption, fruit and vegetable, processed meat intake, oily fish, sleep, physical activity, sedentary behaviours and waist circumference.

In men, after correction for multiple testing, height was positively associated with increased risk of incident cancer overall and at five sites (lung, lymphatic, non-Hodgkins lymphoma, melanoma and leukaemia). The hazard ratios of these associations per 5-cm higher height ranged between 1.01 and 1.03 (Fig. 1). Similar magnitudes of association were observed in women for breast cancer, melanoma, lymphatic and non-Hodgkin lymphoma and all-cause cancer (Fig. 2). For cancer mortality, the associations became non-significant after controlling for multiple testing (Fig. 1). For women, only all-cause cancer remained significant after controlling for multiple comparisons (Fig. 2). The associations were similar in the three models studied (Supplementary Tables S2 and S3).

### Sex differences in the association of height and cancer

**Fig. 3:**
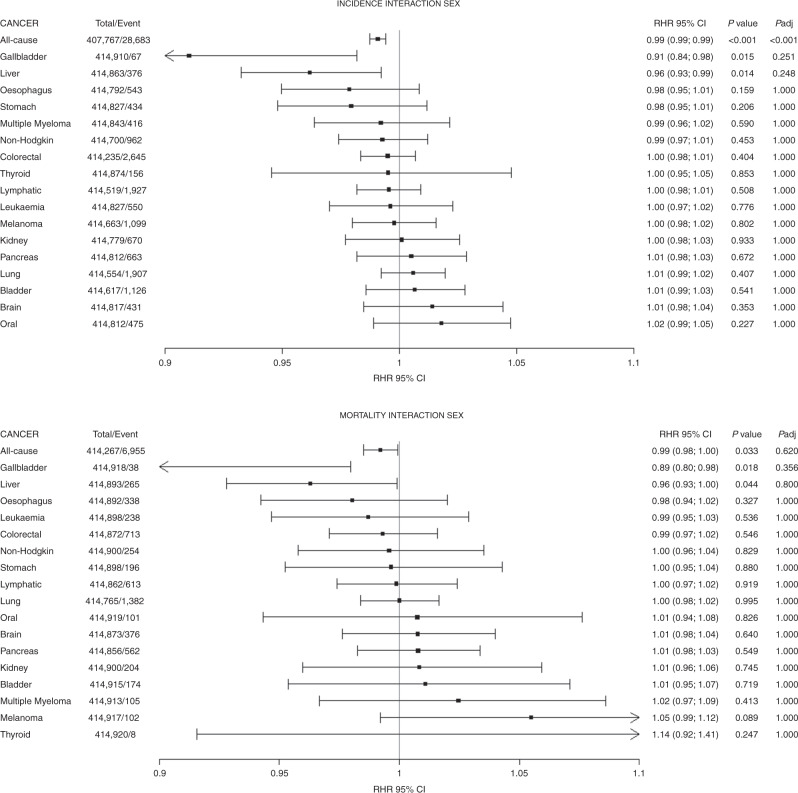
Ratios of HR of men to women for the association of height with incidence and mortality of 18 cancer sites. Data presented as the ratio of HR (interaction terms sex and height) and their 95% CI per 5-cm increment in height. Models were adjusted for age, ethnicity, deprivation index, comorbidity, smoking, alcohol consumption, fruit and vegetable, processed meat intake, oil and fish, sleep, physical activity, sedentary behaviours and waist circumference.

When adjusted for multiple testing, compared to women, men had a weaker association between height and incident all-cause cancer (HR: 0.99, 95% CI: 0.99, 0.99). No other differences in cancer risk were found between men and women (Fig. 3).

### Sensitivity analysis

In sensitivity analysis, height was associated with breast cancer incidence in both pre- and postmenopausal women with similar effect sizes (HR 1.02, 95% CI: 1.01, 1.02, and 1.02, 95% CI: 1.00, 1.02, respectively, *P*_interaction_ 0.94) (Supplementary Table S4). Sensitivity analyses by smoking status are presented in Supplementary Table S5. Height was associated with lung cancer in current and ex-smokers (*P*_interaction_ 0.72). Although similar hazard ratios were observed for smokers and non-smokers, these associations were not significant. Height was associated with liver cancer mortality only among women; no differences were found between those who consumed < or ≥ once a week (Supplementary Table S6).

When the association between height and cancer was further stratified by tall versus short individuals, no differences were observed among men for leukaemia, melanoma, non-Hodgkin lymphoma, lung and all-cause cancer (Supplementary Table S7). The association was lost when the analyses were adjusted for multiple testing (Supplementary Table S7).

### Moderator analysis by IGF-1

**Fig. 4:**
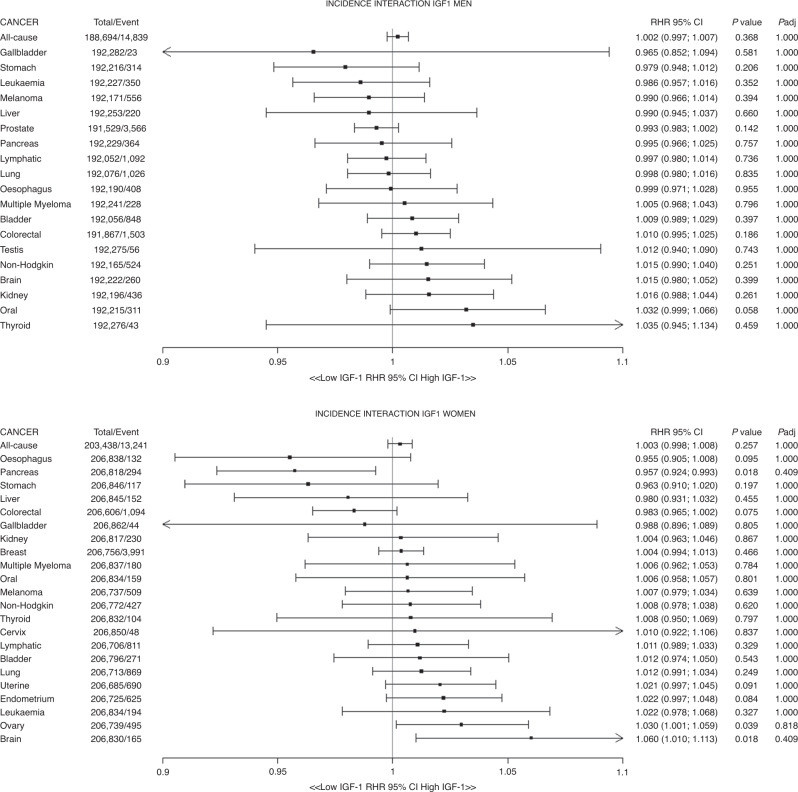
Ratio of HR of low-to-high IGF-1 level for the association of height with incidence of 24 cancer sites of men and women. Data presented as the hazard ratio (interaction term of IGF-1 level and height) and their 95% CI per 5-cm increment in height. Models were adjusted for age, ethnicity, deprivation index, comorbidity, smoking, alcohol consumption, fruit and vegetable, processed meat intake, oil and fish, sleep, physical activity, sedentary behaviours and waist circumference.

For men, the interaction between IGF-1 and cancer incidence is presented in Fig. 4. IGF-1 did not modify the association between height and cancer incidence. HR of height among high IGF-1 group is presented in Supplementary Table S8. Similar results were found for women; no difference in the association was shown by IGF-1 levels (Fig. 4 and Supplementary Fig. S4, Table S9).

## Discussion

The main findings of this study corroborate the associations of height with increased incidence risk of different cancer sites and overall cancer in men and women. However, some of these associations differ by sex. Women have a higher-incidence risk than men for all-cause cancer. We also provide evidence that circulating concentrations of IGF-1 do not modify the association between height and cancer incidence and mortality.

Our findings are in line with previous prospective cohort evidence that suggests that height is associated with a higher risk of postmenopausal breast cancer, melanoma, non-Hodgkin lymphoma, lymphatic cancer and leukaemia.^5,6,8^ However, this study did not corroborate the association of height with colorectal, ovary, endometrium pancreas, prostate and kidney cancer. However, the magnitude and direction of the associations observed in our study agreed with previous evidence.^8^ Therefore, the lack of significant association may be related to a lack of power rather than a lack of association. For all-cause cancer, Choi et al. found HR: 1.088 (95% CI: 1.086, 1.090), similar to our results: HR: 1.008 (95% CI: 1.005, 1.010) for men, 1.010 (95% CI: 1.008, 1.013) for women.

Although our findings are in agreement with evidence derived from prospective studies,^5,6,8^ these associations do not imply causality. An MR study reported a positive association for 17 cancer sites, only six (kidney, non-Hodgkin, colorectal, lung, melanoma and breast cancer) of these cancer sites were significantly associated with height.^22^ Another MR study conducted in UK Biobank participants found a positive association between height and colorectal, endometrium and ovary cancer.^3^ We did observe an association with colorectal cancer in minimally adjusted models; however, no associations were observed for ovary and endometrium cancer in the fully adjusted models. Besides, the magnitude of the association reported by our study was smaller than that reported by MR studies.^3,22^ These differences between studies may be related to residual confounding or reverse causality, as well as effect sizes from MR studies representing a lifelong cumulative risk or lifelong exposure.

The evidence behind the associations of height with non-Hodgkin lymphoma, melanoma and leukaemia has been controversial. However, MR studies have provided evidence that the link between height and these cancers is causal. However, the exact mechanism behind these associations has not been fully elucidated. Some hypotheses suggest that genetic or early environment exposures may play a role in the link between height and these cancers.^23^ Height during adulthood may reflect cumulative exposure to hormones/growth factors and nutritional status in early life.^5^ However, it is biologically plausible that height may indirectly influence carcinogenesis through IGF-1 or immune pathways. The IGF-1 pathways could be triggered by overnutrition, particularly by higher intake of energy-dense foods.^24^ Moreover, recent studies have provided evidence that supports an association between IGF-1 and the risk of several cancers.^9,25,26^ IGF-1 concentration is an important determinant of height and may be a determinant of organ size, and thus IGF-1 could be related to cancer through greater cell division.^27^ Moreover, taller individuals have a greater number of cells that are susceptible to conversion into neoplastic cells.^28^ However, more research is necessary to understand the underlying mechanism.

### Strengths and limitations

The strength of the paper is the comprehensive analysis of height and cancer stratified by sex, in one large cohort. Observational studies cannot determine causality, with confounding and reverse causation. However, to minimise the effect of reverse causation in our study, we excluded all participants with a self-reported medical history of cancer at baseline and those new cancer diagnoses within the first 2 years of follow-up. UK Biobank is not representative of the general population; therefore, caution should be taken in generalising summary statistics to the general population, but estimates of the magnitude of the associations are generalisable.^29^ Some of the sample sizes for cancer-specific sites were small (less than 30); therefore, the association should be interpreted with caution. Our hazard estimate compared to MR study results shares a similar trend but a smaller magnitude of the association.^3,22^ However, we did not find an association between height and cancers of the ovary and endometrium. Some of the conflicting results could be explained by a lack of power for some rare cancers with wide confidence intervals in our study and the MR evidence.

## Conclusion

Height was associated with an increased risk of several cancer sites in men and women. However, the association with all-cause cancer was stronger in women than in men. IGF-1 does not modify the associations between height and cancer risk.

## Supplementary information

Supplementary material: Does insulin-like growth factor moderate the association between height and risk of cancer at 24 sites?

## Data Availability

Data are available upon request from the UK Biobank. Information about data access is available online http://www.ukbiobank.ac.uk/wp-content/uploads/2011/11/UK-Biobank-Protocol.pdf.
