# Supplementary material for: Does insulin-like growth factor moderate the association between height and risk of cancer at 24 sites?
Source: Br J Cancer. 2020 Sep 14;123(11):1697–704. doi: 10.1038/s41416-020-01059-1 (PMC7686481; doi:10.1038/s41416-020-01059-1)
Supplement: Supplementary file 1 — Supplementary material: Does insulin-like growth factor moderate the association between height and risk of cancer at 24 sites? [file 41416_2020_1059_MOESM1_ESM.docx]

Supplementary material: Does insulin-like growth factor moderate the association between height and risk of cancer at 24 sites?


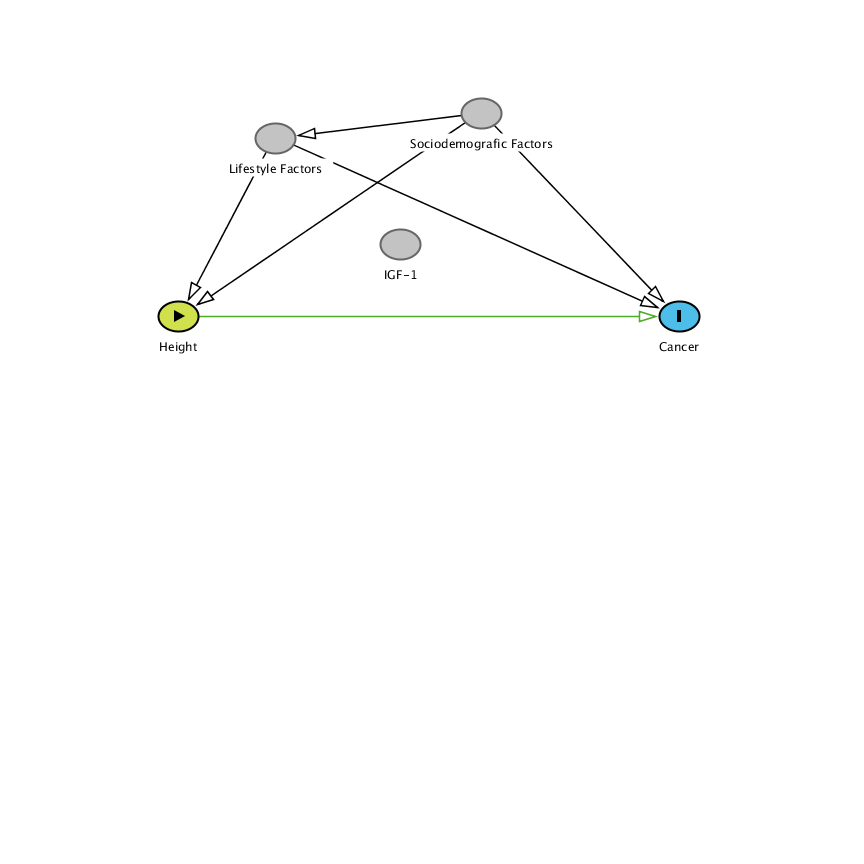


Supplementary Figure S1: Directed Acyclic Graphs Potential confounders identified a priori based on established relationships, with cancer and height.

Sociodemographic factors: age, ethnicity, deprivation, Lifestyle factors: diet (fruits and vegetables, oily fish, alcohol, red and process meat intake), sleep, physical activity and sedentary behaviour.

**Supplementary Table S1**: Person Correlation IGF-1 and height by age and sex

|  | **Both sex** |  | **Men** |  | **Women** |  |
| --- | --- | --- | --- | --- | --- | --- |
| **Height and IGF-1** | **Coefficient** | **P value** | **Coefficient** | **P value** | **Coefficient** | **P value** |
| Overall | 0.133 | <0.001 | 0.118 | <0.001 | 0.107 | <0.001 |
| <45 years | 0.038 | <0.001 | 0.106 | <0.001 | 0.070 | <0.001 |
| 45-50 years | 0.083 | <0.001 | 0.108 | <0.001 | 0.080 | <0.001 |
| 50-55 years | 0.116 | <0.001 | 0.095 | <0.001 | 0.070 | <0.001 |
| 55-60 years | 0.119 | <0.001 | 0.084 | <0.001 | 0.050 | <0.001 |
| 60-65 years | 0.130 | <0.001 | 0.082 | <0.001 | 0.047 | <0.001 |
| >65 years | 0.136 | <0.001 | 0.066 | <0.001 | 0.034 | <0.001 |
| >70 years | 0.128 | <0.001 | 0.028 | 0.389 | 0.016 | 0.628 |


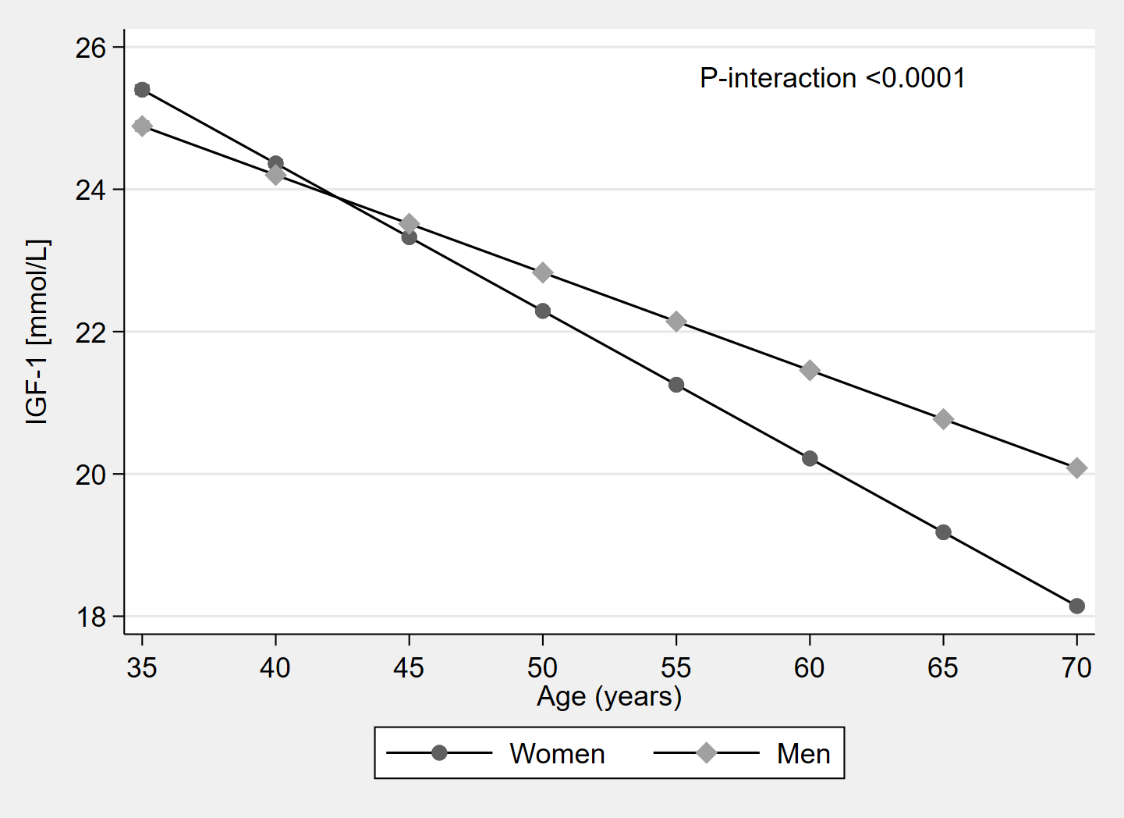


**Supplementary Figure S2:** IGF-1 blood concentration by age and sex.


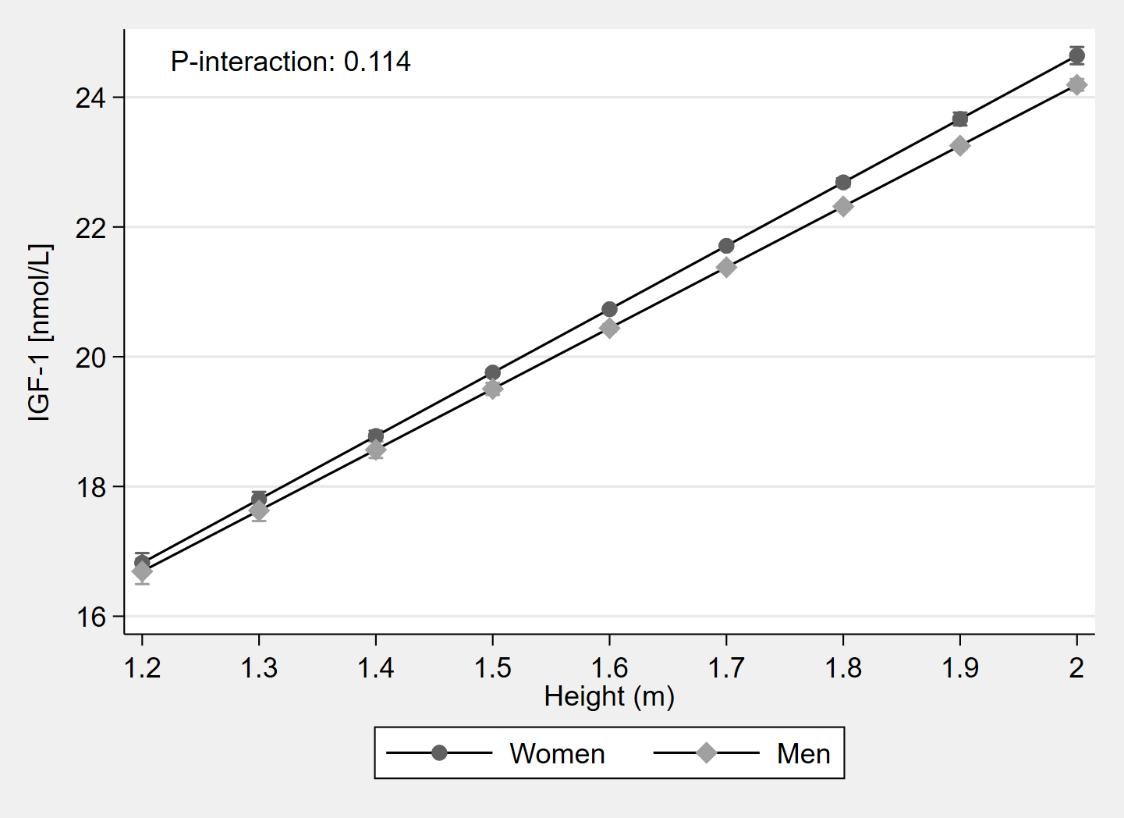


**Supplementary Figure S3:** IGF-1 blood concentration by height and sex.

**Supplementary Table S2:** Association height (per 5 cm) and cancer risk incidence, men and women.

| **Site cancer** | **Total** | **Event** | **Model 1** | **P-value** | P_adjusted_ | **Model 2** | **P-value** | P_adjusted_ | **Model 3** | **P-value** | P_adjusted_ |
| --- | --- | --- | --- | --- | --- | --- | --- | --- | --- | --- | --- |
| **MEN** | | | | | | | | | | | |
| All-cause | 188,694 | 14,839 | **1.01 (1.01; 1.01)** | <0.001 | <0.001 | **1.01 (1.01; 1.01)** | <0.001 | <0.001 | **1.01 (1.01; 1.01)** | <0.001 | <0.001 |
| Bladder | 192,056 | 848 | 1.01 (1.00; 1.02) | 0.288 | 1.00 | 1.01 (1.00; 1.02) | 0.230 | 1.00 | 1.00 (0.99; 1.01) | 0.492 | 1.00 |
| Brain | 192,222 | 260 | 1.01 (0.99; 1.03) | 0.241 | 1.00 | 1.01 (0.99; 1.03) | 0.254 | 1.00 | 1.01 (0.99; 1.03) | 0.204 | 1.00 |
| Colorectal | 191,867 | 1,503 | 1.00 (1.00; 1.01) | 0.360 | 1.00 | 1.00 (1.00; 1.01) | 0.378 | 1.00 | 1.00 (0.99; 1.01) | 0.835 | 1.00 |
| Gallbladder | 192,282 | 23 | 0.95 (0.89; 1.01) | 0.094 | 1.00 | 0.95 (0.89; 1.01) | 0.083 | 1.00 | 0.94 (0.88; 1.00) | 0.057 | 0.741 |
| Kidney | 192,196 | 436 | 1.00 (0.99; 1.02) | 0.484 | 1.00 | 1.01 (0.99; 1.02) | 0.347 | 1.00 | 1.00 (0.99; 1.02) | 0.852 | 1.00 |
| Leukaemia | 192,227 | 350 | **1.03 (1.01; 1.05)** | <0.001 | <0.001 | **1.03 (1.01; 1.05)** | <0.001 | <0.001 | **1.03 (1.01; 1.05)** | <0.001 | <0.001 |
| Liver | 192,253 | 220 | 0.99 (0.97; 1.01) | 0.405 | 1.00 | 0.99 (0.97; 1.01) | 0.552 | 1.00 | 0.99 (0.97; 1.01) | 0.205 | 1.00 |
| Lung | 192,076 | 1,026 | 1.01 (1.00; 1.02) | 0.069 | 0.897 | **1.01 (1.00; 1.02)** | 0.005 | 0.075 | **1.02 (1.01; 1.02)** | 0.001 | 0.016 |
| Lymphatic | 192,052 | 1,092 | **1.02 (1.01; 1.03)** | <0.001 | <0.001 | **1.02 (1.01; 1.03)** | <0.001 | <0.001 | **1.02 (1.01; 1.03)** | <0.001 | <0.001 |
| Melanoma | 192,171 | 556 | **1.03 (1.02; 1.04)** | <0.001 | <0.001 | **1.03 (1.02; 1.04)** | <0.001 | <0.001 | **1.03 (1.01; 1.04)** | <0.001 | <0.001 |
| Multiple Myeloma | 192,241 | 228 | 1.01 (0.99; 1.03) | 0.212 | 1.00 | 1.01 (0.99; 1.03) | 0.215 | 1.00 | 1.01 (0.99; 1.03) | 0.262 | 1.00 |
| Non-Hodgkin | 192,165 | 524 | **1.02 (1.01; 1.03)** | 0.002 | 0.032 | **1.02 (1.01; 1.03)** | 0.001 | 0.016 | **1.02 (1.01; 1.04)** | 0.001 | 0.016 |
| Oesophagus | 192,190 | 408 | 0.98 (0.97; 1.00) | 0.039 | 0.585 | 0.99 (0.97; 1.00) | 0.097 | 1.00 | 0.98 (0.97; 1.00) | 0.010 | 0.140 |
| Oral | 192,215 | 311 | 1.00 (0.98; 1.01) | 0.795 | 1.00 | 1.00 (0.98; 1.02) | 0.872 | 1.00 | 1.00 (0.98; 1.02) | 0.919 | 1.00 |
| Pancreas | 192,229 | 364 | **1.02 (1.00; 1.03)** | 0.043 | 0.602 | 1.02 (1.00; 1.03) | 0.052 | 0.728 | 1.01 (1.00; 1.03) | 0.105 | 1.00 |
| Prostate | 191,529 | 3,566 | 1.00 (1.00; 1.01) | 0.357 | 1.00 | 1.00 (1.00; 1.01) | 0.500 | 1.00 | 1.00 (1.00; 1.01) | 0.243 | 1.00 |
| Stomach | 192,216 | 314 | 1.00 (0.98; 1.01) | 0.597 | 1.00 | 1.00 (0.98; 1.01) | 0.802 | 1.00 | 0.99 (0.97; 1.01) | 0.279 | 1.00 |
| Testis | 192,275 | 56 | 1.04 (1.00; 1.08) | 0.076 | 0.912 | 1.03 (1.00; 1.07) | 0.080 | 1.00 | 1.03 (1.00; 1.08) | 0.082 | 0.984 |
| Thyroid | 192,276 | 43 | 1.02 (0.97; 1.06) | 0.415 | 1.00 | 1.01 (0.97; 1.06) | 0.556 | 1.00 | 1.01 (0.97; 1.06) | 0.594 | 1.00 |
| **WOMEN** | | | | | | | | | | | |
| All-cause | 219,073 | 13,844 | **1.01 (1.01; 1.01)** | <0.001 | <0.001 | **1.01 (1.01; 1.01)** | <0.001 | <0.001 | **1.01 (1.01; 1.01)** | <0.001 | <0.001 |
| Bladder | 222,561 | 278 | 1.00 (0.98; 1.02) | 0.940 | 1.00 | 1.00 (0.98; 1.02) | 0.899 | 1.00 | 1.00 (0.98; 1.02) | 0.842 | 1.00 |
| Brain | 222,595 | 171 | 1.00 (0.98; 1.03) | 0.887 | 1.00 | 1.00 (0.98; 1.02) | 0.993 | 1.00 | 1.00 (0.98; 1.02) | 0.988 | 1.00 |
| Breast | 221,450 | 4,283 | **1.02 (1.01; 1.02)** | <0.001 | <0.001 | **1.02 (1.01; 1.02)** | <0.001 | <0.001 | **1.01 (1.01; 1.02)** | <0.001 | <0.001 |
| Cervix | 222,613 | 50 | 0.98 (0.93; 1.02) | 0.292 | 1.00 | 0.98 (0.94; 1.03) | 0.413 | 1.00 | 0.98 (0.94; 1.02) | 0.377 | 1.00 |
| Colorectal | 222,368 | 1,142 | 1.01 (1.00; 1.02) | 0.193 | 1.00 | 1.01 (1.00; 1.02) | 0.116 | 1.00 | 1.01 (1.00; 1.02) | 0.186 | 1.00 |
| Endometrium | 222,491 | 646 | 0.99 (0.98; 1.00) | 0.242 | 1.00 | 1.00 (0.98; 1.01) | 0.500 | 1.00 | 0.99 (0.98; 1.00) | 0.056 | 0.952 |
| Gallbladder | 222,628 | 44 | 1.03 (0.98; 1.08) | 0.280 | 1.00 | 1.03 (0.98; 1.08) | 0.236 | 1.00 | 1.03 (0.98; 1.08) | 0.298 | 1.00 |
| Kidney | 222,583 | 234 | 1.00 (0.98; 1.02) | 0.881 | 1.00 | 1.00 (0.98; 1.02) | 0.777 | 1.00 | 1.00 (0.98; 1.02) | 0.882 | 1.00 |
| Leukaemia | 222,600 | 200 | 1.03 (1.01; 1.06) | 0.004 | 0.072 | 1.03 (1.01; 1.06) | 0.003 | 0.054 | 1.03 (1.01; 1.06) | 0.004 | 0.072 |
| Liver | 222,610 | 156 | 1.02 (1.00; 1.05) | 0.115 | 1.00 | 1.02 (1.00; 1.05) | 0.072 | 1.00 | 1.02 (1.00; 1.05) | 0.086 | 1.00 |
| Lung | 222,478 | 881 | 1.00 (0.99; 1.01) | 0.719 | 1.00 | 1.00 (0.99; 1.02) | 0.399 | 1.00 | 1.00 (0.99; 1.01) | 0.468 | 1.00 |
| Lymphatic | 222,467 | 835 | **1.03 (1.01; 1.04)** | <0.001 | <0.001 | **1.03 (1.02; 1.04)** | <0.001 | <0.001 | **1.03 (1.02; 1.04)** | <0.001 | <0.001 |
| Melanoma | 222,492 | 543 | **1.02 (1.01; 1.04)** | 0.001 | 0.019 | **1.02 (1.01; 1.04)** | 0.001 | 0.019 | **1.02 (1.01; 1.04)** | 0.001 | 0.019 |
| Multiple Myeloma | 222,602 | 188 | 1.02 (1.00; 1.04) | 0.111 | 1.00 | 1.02 (1.00; 1.05) | 0.050 | 0.800 | 1.02 (1.00; 1.04) | 0.071 | 1.00 |
| Non-Hodgkin | 222,535 | 438 | **1.03 (1.01; 1.04)** | <0.001 | <0.001 | **1.03 (1.01; 1.05)** | <0.001 | <0.001 | **1.03 (1.01; 1.05)** | <0.001 | <0.001 |
| Oesophagus | 222,602 | 135 | 1.01 (0.98; 1.04) | 0.544 | 1.00 | 1.01 (0.98; 1.04) | 0.494 | 1.00 | 1.01 (0.98; 1.04) | 0.469 | 1.00 |
| Oral | 222,597 | 164 | 0.98 (0.96; 1.01) | 0.185 | 1.00 | 0.98 (0.96; 1.01) | 0.172 | 1.00 | 0.98 (0.96; 1.01) | 0.204 | 1.00 |
| Ovary | 222,502 | 515 | 1.01 (0.99; 1.02) | 0.355 | 1.00 | 1.01 (0.99; 1.02) | 0.318 | 1.00 | 1.01 (0.99; 1.02) | 0.306 | 1.00 |
| Pancreas | 222,583 | 299 | 1.01 (0.99; 1.03) | 0.211 | 1.00 | 1.01 (0.99; 1.03) | 0.204 | 1.00 | 1.01 (0.99; 1.03) | 0.260 | 1.00 |
| Stomach | 222,611 | 120 | 1.01 (0.98; 1.04) | 0.392 | 1.00 | 1.01 (0.99; 1.04) | 0.320 | 1.00 | 1.01 (0.98; 1.04) | 0.378 | 1.00 |
| Thyroid | 222,598 | 113 | 1.03 (1.00; 1.06) | 0.062 | 1.00 | 1.03 (1.00; 1.06) | 0.047 | 0.799 | 1.03 (1.00; 1.06) | 0.077 | 1.00 |
| Uterine | 222,448 | 713 | 0.99 (0.98; 1.01) | 0.285 | 1.00 | 1.00 (0.99; 1.01) | 0.582 | 1.00 | 0.99 (0.98; 1.00) | 0.072 | 1.00 |

Data is presented as hazard ratio (HR) and their 95% confidence interval (95% CI) in men and women, P_adjusted_: P-value adjusted for multiple testing

Model 1: Adjusted for age, ethnicity, deprivation index and comorbidity.

Model 2; Adjusted for model 1 plus smoking, alcohol consumption, fruit & vegetable, processed meat intake, oily fish, sleep, physical activity and sedentary behaviours.

Model 3: Adjusted for model 2 plus waist circumference.

Supplementary Table S3: Association height (per 5 cm) and cancer risk mortality, men and women.

| **Site cancer** | **Total** | **Event** | **Model 1** | **P-value** | P_adjusted_ | **Model 2** | **P-value** | P_adjusted_ | **Model 3** | **P-value** | P_adjusted_ |
| --- | --- | --- | --- | --- | --- | --- | --- | --- | --- | --- | --- |
| **MEN** | | | | | | | | | | | |
| All-cause | 191,881 | 3,986 | **1.01 (1.00; 1.01)** | 0.019 | 0.361 | **1.01 (1.00; 1.01)** | 0.001 | 0.019 | 1.01 (1.00; 1.01) | 0.013 | 0.247 |
| Bladder | 192,284 | 139 | 1.01 (0.98; 1.03) | 0.522 | 1.00 | 1.01 (0.99; 1.04) | 0.432 | 1.00 | 1.01 (0.98; 1.03) | 0.536 | 1.00 |
| Brain | 192,254 | 231 | 1.01 (0.99; 1.03) | 0.560 | 1.00 | 1.01 (0.99; 1.02) | 0.592 | 1.00 | 1.01 (0.99; 1.03) | 0.470 | 1.00 |
| Colorectal | 192,254 | 417 | 1.01 (1.00; 1.02) | 0.200 | 1.00 | 1.01 (0.99; 1.02) | 0.211 | 1.00 | 1.00 (0.99; 1.02) | 0.654 | 1.00 |
| Gallbladder | 192,284 | 14 | 0.93 (0.86; 1.00) | 0.059 | 1.00 | 0.93 (0.86; 1.00) | 0.056 | 0.952 | 0.92 (0.85; 1.00) | 0.038 | 0.570 |
| Kidney | 192,273 | 153 | 1.00 (0.98; 1.02) | 0.981 | 1.00 | 1.00 (0.98; 1.03) | 0.797 | 1.00 | 1.00 (0.97; 1.02) | 0.878 | 1.00 |
| Leukaemia | 192,275 | 160 | 1.02 (1.00; 1.04) | 0.082 | 1.00 | 1.02 (1.00; 1.05) | 0.064 | 0.96 | 1.02 (1.00; 1.05) | 0.062 | 0.868 |
| Liver | 192,272 | 155 | 1.00 (0.98; 1.02) | 0.928 | 1.00 | 1.00 (0.98; 1.03) | 0.754 | 1.00 | 1.00 (0.97; 1.02) | 0.720 | 1.00 |
| Lung | 192,194 | 790 | 1.01 (1.00; 1.02) | 0.295 | 1.00 | 1.01 (1.00; 1.02) | 0.058 | 0.952 | 1.01 (1.00; 1.02) | 0.018 | 0.324 |
| Lymphatic | 192,250 | 380 | 1.02 (1.00; 1.03) | 0.024 | 0.432 | 1.02 (1.00; 1.03) | 0.013 | 0.234 | 1.02 (1.00; 1.03) | 0.020 | 0.340 |
| Melanoma | 192,283 | 64 | 1.02 (0.98; 1.05) | 0.418 | 1.00 | 1.02 (0.98; 1.05) | 0.384 | 1.00 | 1.02 (0.98; 1.05) | 0.417 | 1.00 |
| Multiple Myeloma | 192,282 | 54 | 1.03 (0.99; 1.07) | 0.114 | 1.00 | 1.03 (0.99; 1.07) | 0.126 | 1.00 | 1.03 (0.99; 1.07) | 0.175 | 1.00 |
| Non-Hodgkin | 192,271 | 157 | 1.01 (0.98; 1.03) | 0.525 | 1.00 | 1.01 (0.99; 1.03) | 0.365 | 1.00 | 1.01 (0.99; 1.03) | 0.414 | 1.00 |
| Oesophagus | 192,263 | 262 | 0.98 (0.97; 1.00) | 0.080 | 1.00 | 0.99 (0.97; 1.01) | 0.182 | 1.00 | 0.98 (0.96; 1.00) | 0.025 | 0.400 |
| Oral | 192,285 | 73 | 1.00 (0.97; 1.04) | 0.811 | 1.00 | 1.01 (0.98; 1.04) | 0.620 | 1.00 | 1.01 (0.98; 1.05) | 0.427 | 1.00 |
| Pancreas | 192,246 | 306 | 1.01 (1.00; 1.03) | 0.102 | 1.00 | 1.01 (1.00; 1.03) | 0.110 | 1.00 | 1.01 (0.99; 1.03) | 0.237 | 1.00 |
| Prostate | 192,273 | 342 | 1.01 (1.00; 1.03) | 0.150 | 1.00 | 1.01 (1.00; 1.03) | 0.122 | 1.00 | 1.01 (0.99; 1.03) | 0.268 | 1.00 |
| Stomach | 192,270 | 131 | 1.01 (0.99; 1.04) | 0.400 | 1.00 | 1.02 (0.99; 1.04) | 0.238 | 1.00 | 1.01 (0.98; 1.04) | 0.474 | 1.00 |
| Testis | 192,273 | 0 | NA. |  | 1.00 | NA. |  | 1.00 | NA. |  | 1.00 |
| Thyroid | 192,288 | 5 | 0.99 (0.87; 1.13) | 0.903 | 1.00 | 0.99 (0.86; 1.13) | 0.843 | 1.00 | 0.97 (0.85; 1.11) | 0.705 | 0.247 |
| **WOMEN** | | | | | | | | | | | |
| All-cause | 222,386 | 2,969 | **1.01 (1.00; 1.02)** | 0.001 | 0.023 | **1.01 (1.01; 1.02)** | <0.001 | <0.001 | **1.01 (1.00; 1.02)** | 0.001 | 0.023 |
| Bladder | 222,631 | 35 | 1.00 (0.95; 1.06) | 0.857 | 1.00 | 1.01 (0.95; 1.06) | 0.811 | 1.00 | 1.00 (0.95; 1.06) | 0.954 | 1.00 |
| Brain | 222,619 | 145 | 1.00 (0.98; 1.03) | 0.817 | 1.00 | 1.00 (0.98; 1.03) | 0.857 | 1.00 | 1.00 (0.98; 1.03) | 0.819 | 1.00 |
| Breast | 222,621 | 272 | 1.02 (1.00; 1.04) | 0.025 | 0.550 | 1.02 (1.00; 1.04) | 0.035 | 0.735 | 1.02 (1.00; 1.04) | 0.055 | 1.00 |
| Cervix | 222,633 | 10 | 0.96 (0.87; 1.06) | 0.373 | 1.00 | 0.96 (0.87; 1.06) | 0.457 | 1.00 | 0.97 (0.88; 1.07) | 0.518 | 1.00 |
| Colorectal | 222,618 | 296 | 1.01 (0.99; 1.03) | 0.188 | 1.00 | 1.01 (1.00; 1.03) | 0.123 | 1.00 | 1.01 (0.99; 1.03) | 0.156 | 1.00 |
| Endometrium | 222,633 | 69 | 1.01 (0.97; 1.05) | 0.750 | 1.00 | 1.01 (0.97; 1.05) | 0.634 | 1.00 | 1.00 (0.96; 1.04) | 0.997 | 1.00 |
| Gallbladder | 222,634 | 24 | 1.02 (0.96; 1.09) | 0.519 | 1.00 | 1.02 (0.96; 1.09) | 0.476 | 1.00 | 1.02 (0.96; 1.09) | 0.544 | 1.00 |
| Kidney | 222,627 | 51 | 0.99 (0.94; 1.03) | 0.568 | 1.00 | 0.99 (0.94; 1.03) | 0.594 | 1.00 | 0.98 (0.94; 1.03) | 0.405 | 1.00 |
| Leukaemia | 222,623 | 78 | 1.03 (1.00; 1.07) | 0.080 | 1.00 | 1.03 (1.00; 1.07) | 0.080 | 1.00 | 1.03 (0.99; 1.07) | 0.111 | 1.00 |
| Liver | 222,621 | 110 | 1.03 (1.00; 1.06) | 0.046 | 0.966 | 1.03 (1.00; 1.07) | 0.029 | 0.638 | 1.03 (1.00; 1.06) | 0.043 | 0.946 |
| Lung | 222,571 | 592 | 1.00 (0.99; 1.02) | 0.604 | 1.00 | 1.01 (0.99; 1.02) | 0.279 | 1.00 | 1.01 (0.99; 1.02) | 0.356 | 1.00 |
| Lymphatic | 222,612 | 233 | 1.02 (1.00; 1.04) | 0.117 | 1.00 | 1.02 (1.00; 1.04) | 0.073 | 1.00 | 1.02 (1.00; 1.04) | 0.114 | 1.00 |
| Melanoma | 222,634 | 38 | 0.96 (0.91; 1.01) | 0.091 | 1.00 | 0.96 (0.91; 1.01) | 0.105 | 1.00 | 0.96 (0.91; 1.01) | 0.114 | 1.00 |
| Multiple Myeloma | 222,631 | 51 | 1.01 (0.97; 1.06) | 0.560 | 1.00 | 1.02 (0.98; 1.07) | 0.400 | 1.00 | 1.02 (0.97; 1.06) | 0.479 | 1.00 |
| Non-Hodgkin | 222,629 | 97 | 1.01 (0.98; 1.04) | 0.603 | 1.00 | 1.01 (0.98; 1.04) | 0.495 | 1.00 | 1.01 (0.98; 1.04) | 0.572 | 1.00 |
| Oesophagus | 222,629 | 76 | 1.01 (0.97; 1.05) | 0.650 | 1.00 | 1.01 (0.97; 1.05) | 0.584 | 1.00 | 1.01 (0.98; 1.05) | 0.535 | 1.00 |
| Oral | 222,634 | 28 | 1.00 (0.94; 1.06) | 0.959 | 1.00 | 1.01 (0.95; 1.07) | 0.858 | 1.00 | 1.01 (0.95; 1.07) | 0.832 | 1.00 |
| Ovary | 222,622 | 246 | 1.01 (0.99; 1.03) | 0.573 | 1.00 | 1.01 (0.99; 1.03) | 0.458 | 1.00 | 1.01 (0.99; 1.03) | 0.441 | 1.00 |
| Pancreas | 222,610 | 256 | 1.01 (0.99; 1.03) | 0.564 | 1.00 | 1.01 (0.99; 1.03) | 0.542 | 1.00 | 1.00 (0.99; 1.03) | 0.634 | 1.00 |
| Stomach | 222,628 | 65 | 1.01 (0.97; 1.05) | 0.544 | 1.00 | 1.02 (0.98; 1.06) | 0.417 | 1.00 | 1.01 (0.97; 1.05) | 0.529 | 1.00 |
| Thyroid | 222,632 | 3 | 0.89 (0.75; 1.07) | 0.218 | 1.00 | 0.89 (0.75; 1.06) | 0.180 | 1.00 | 0.88 (0.74; 1.05) | 0.152 | 1.00 |
| Uterine | 222,630 | 104 | 1.01 (0.98; 1.04) | 0.680 | 1.00 | 1.01 (0.98; 1.04) | 0.500 | 1.00 | 1.00 (0.97; 1.04) | 0.773 | 1.00 |

Data is presented as hazard ratio (HR) and their 95% confidence interval (95% CI) in men and women, P_adjusted_: P-value adjusted for multiple testing.

Model 1: Adjusted for age, sex ethnicity, deprivation index and comorbidity.

Model 2; Adjusted for model 1 plus smoking, alcohol consumption, fruit & vegetable, processed meat intake, oily fish, sleep, physical activity and sedentary behaviours.

Model 3: Adjusted for model 2 plus waist circumference.

**Supplementary Table S4:** Association height (per 5 cm) and breast, cervix, endometrium, ovary and uterine cancer incidence and mortality by menopausal status.

|  | **Overall** | **Postmenopausal** | **Premenopausal** | **P interaction** |
| --- | --- | --- | --- | --- |
| **Breast** | | | |  |
| Total/event | 221,445/4,283 | 131,976/2,744 | 55,743/883 |  |
| HR (95% CI) | **1.01 (1.01; 1.02)** | **1.02 (1.01; 1.02)** | **1.02 (1.01; 1.02)** | 0.936 |
| Total/deaths | 222,616/272 | 132,751/180 | 55,956/56 |  |
| HR (95% CI) | 1.02 (1.00; 1.04) | 1.02 (0.99; 1.04) | 1.02 (0.99; 1.04) | 0.509 |
| **Cervix** | | | |  |
| Total/event | 222,608/50 | 132,745/34 | 55,952/13 |  |
| HR (95% CI) | 0.98 (0.94; 1.02) | 0.98 (0.93; 1.03) | 0.98 (0.93; 1.03) | 0.774 |
| Total/deaths | 222,628/10 | 132,759/6 | 55,957/3 |  |
| HR (95% CI) | 0.97 (0.88; 1.07) | 0.97 (0.85; 1.10) | 0.97 (0.85; 1.10) | 0.621 |
| **Endometrium** | | | |  |
| Total/event | 222,486/646 | 132,638/526 | 55,944/94 |  |
| HR (95% CI) | 0.99 (0.98; 1.00) | 0.99 (0.98; 1.01) | 0.99 (0.98; 1.01) | 0.150 |
| Total/deaths | 222,628/69 | 132,758/62 | 0,001/1 |  |
| HR (95% CI) | 1.00 (0.96; 1.04) | 1.00 (0.96; 1.04) | 1.00 (0.96; 1.04) | 0.931 |
| **Ovary** | | | |  |
| Total/event | 222,497/515 | 132,663/380 | 55,940/78 |  |
| HR (95% CI) | 1.01 (0.99; 1.02) | 1.00 (0.99; 1.02) | 1.00 (0.99; 1.02) | 0.953 |
| Total/deaths | 222,617/246 | 132,749/197 | 55,956/21 |  |
| HR (95% CI) | 1.01 (0.99; 1.03) | 1.01 (0.98; 1.03) | 1.01 (0.98; 1.03) | 0.563 |
| **Uterine** | | | |  |
| Total/event | 222,443/713 | 132,607/574 | 55,934/111 |  |
| HR (95% CI) | 0.99 (0.98; 1.00) | 0.99 (0.98; 1.01) | 0.99 (0.98; 1.01) | 0.249 |
| Total/deaths | 222,625/104 | 132,756/89 | 55,957/9 |  |
| HR (95% CI) | 1.00 (0.97; 1.04) | 1.01 (0.97; 1.04) | 1.01 (0.97; 1.04) | 0.801 |

_Data is presented as hazard ratio and their 95% intervals in women_

_Model: Adjusted for age, sex ethnicity, deprivation index, comorbidity, smoking, hormone replacement, alcohol consumption, fruit & vegetable, processed meat intake, oily fish, sleep, physical activity, sedentary behaviours and waist circumference. HR: hazard ratio._

**Supplementary Table S5:** Association height (per 5 cm) and lung cancer incidence and mortality by smoking status.

|  | **Overall** | **No smokers** | **Ex-smokers** | **Current smokers** | **P interaction** |
| --- | --- | --- | --- | --- | --- |
| **Lung Incidence** | | | | |  |
| Total/event | 192,076/1,026 | 95,266/104 | 73,286/468 | 23,524/454 |  |
| Men | **1.02 (1.01; 1.02)** | 1.01 (0.98; 1.04) | **1.02 (1.00; 1.03)** | **1.02 (1.00; 1.03)** | 0.720 |
| Total/event | 222,478/881 | 133,818/180 | 69,255/380 | 19,405/321 |  |
| Women | 1.00 (0.99; 1.01) | 1.01 (0.99; 1.03) | 1.00 (0.99; 1.02) | 1.00 (0.98; 1.02) | 0.129 |
| **Lung Mortality** | | | | |  |
| Total/deaths | 192,194/790 | 95,283/68 | 73,353/368 | 23,558/354 |  |
| Men | **1.01 (1.00; 1.02)** | 1.03 (0.99; 1.07) | **1.01 (1.00; 1.03)** | **1.01 (1.00; 1.03)** | 0.870 |
| Total/deaths | 222,571/592 | 133,843/90 | 69,292/270 | 19,436/232 |  |
| Women | 1.01 (0.99; 1.02) | 1.02 (0.99; 1.06) | 1.00 (0.98; 1.02) | 1.00 (0.98; 1.02) | 0.247 |

_Data is presented as hazard ratio and their 95% intervals in men and women_

_Model: Adjusted for age, sex ethnicity, deprivation index, comorbidity, alcohol consumption, fruit & vegetable, processed meat intake, oily fish, sleep, physical activity, sedentary behaviours and waist circumference. HR: Hazard ratio_

**Supplementary Table S6:** Association height (per 5 cm) and liver and stomach cancer incidence and mortality by alcohol risk.

|  | **Overall** | **No risk OH** | **Risk OH** | **P interaction** |
| --- | --- | --- | --- | --- |
| **Liver Incidence** | | | |  |
| Total/event | 192,253/220 | 25,139/49 | 167,114/171 |  |
| Men | 0.99 (0.97; 1.01) | 1.00 (0.96; 1.04) | 0.98 (0.96; 1.01) | 0.700 |
| Total/event | 222,610/156 | 52,950/59 | 169,660/97 |  |
| Women | 1.02 (1.00; 1.05) | 1.03 (0.99; 1.07) | 1.02 (0.98; 1.05) | 0.525 |
| **Liver mortality** | | | |  |
| Total/deaths | 192,272/155 | 25,141/31 | 167,131/124 |  |
| Men | 1.00 (0.97; 1.02) | 1.00 (0.95; 1.05) | 0.99 (0.97; 1.02) | 0.971 |
| Total/deaths | 222,621/110 | 52,952/42 | 169,669/68 |  |
| Women | **1.03 (1.00; 1.06)** | 1.04 (0.99; 1.09) | 1.03 (0.99; 1.07) | 0.553 |
| **Stomach incidence** | | | |  |
| Total/event | 192,216/314 | 25,131/58 | 167,085/256 |  |
| Men | 0.99 (0.97; 1.01) | 0.99 (0.96; 1.03) | 0.99 (0.97; 1.01) | 0.849 |
| Total/event | 222,611/120 | 52,948/40 | 169,663/80 |  |
| Women | 1.01 (0.98; 1.04) | 1.01 (0.96; 1.06) | 1.01 (0.98; 1.05) | 0.984 |
| **Stomach mortality** | | | |  |
| Total/deaths | 192,270/131 | 25,142/33 | 167,128/98 |  |
| Men | 1.01 (0.98; 1.04) | 1.03 (0.98; 1.08) | 1.00 (0.97; 1.03) | 0.463 |
| Total/deaths | 222,628/65 | 52,955/22 | 169,673/43 |  |
| Women | 1.01 (0.97; 1.05) | 1.02 (0.96; 1.09) | 1.00 (0.96; 1.05) | 0.600 |

_Data is presented as hazard ratio and their 95% intervals in men and women_

_Model: Adjusted for age, sex ethnicity, deprivation index, comorbidity, alcohol consumption, fruit & vegetable, processed meat intake, oily fish, sleep, physical activity, sedentary behaviours and waist circumference. OH: alcohol: Alcohol risk unit > once a week._

**Supplementary Table S7:** Association height (per 5 cm) and 24 cancer sites incidence by group of height ≥ or < 162 cm, for women, ≥ or < 176 cm for men.

| Site cancer | Category Height | Total | Event | HR 95% CI | P value | P_adjusted_ | P _interation_ |  | Total | Event | HR 95% CI | P value | P_adjusted_ | P _interation_ | Padj |
| --- | --- | --- | --- | --- | --- | --- | --- | --- | --- | --- | --- | --- | --- | --- | --- |
|  |  |  | Men | | | | |  |  |  | Women |  |  |  |  |
| All-cause | <176m, <162w | 98,433 | 8,101 | **1.01 (1.00; 1.01)** | **0.003** | 0.114 | 0.731 | 1.00 | 102,882 | 6,550 | **1.01 (1.01; 1.02)** | **<0.001** | **<0.001** | 0.242 | 1.00 |
|  | ≥176m, ≥162w | 90,261 | 6,738 | **1.01 (1.00; 1.01)** | **0.013** | 0.455 |  |  | 116,191 | 7,294 | **1.01 (1.00; 1.01)** | **0.024** | 1.00 |  |  |
| Bladder | <176m, <162w | 100,258 | 484 | 1.01 (0.99; 1.03) | 0.594 | 1.00 | 0.643 | 1.00 | 104,516 | 140 | 0.99 (0.95; 1.03) | 0.536 | 1.00 | 0.810 | 1.00 |
|  | ≥176m, ≥162w | 91,798 | 364 | 1.01 (0.99; 1.04) | 0.407 | 1.00 |  |  | 118,045 | 138 | 0.98 (0.94; 1.02) | 0.336 | 1.00 |  |  |
| Brain | <176m, <162w | 100,352 | 134 | 1.02 (0.98; 1.06) | 0.314 | 1.00 | 0.647 | 1.00 | 104,539 | 89 | 1.02 (0.97; 1.08) | 0.389 | 1.00 | 0.691 | 1.00 |
|  | ≥176m, ≥162w | 91,870 | 126 | 1.00 (0.96; 1.04) | 0.962 | 1.00 |  |  | 118,056 | 82 | 1.00 (0.95; 1.06) | 0.922 | 1.00 |  |  |
| Colorectal | <176m, <162w | 100,152 | 819 | 1.00 (0.99; 1.02) | 0.675 | 1.00 | 0.133 | 1.00 | 104,431 | 576 | 1.00 (0.98; 1.02) | 0.747 | 1.00 | 0.284 | 1.00 |
|  | ≥176m, ≥162w | 91,715 | 684 | 0.99 (0.97; 1.00) | 0.133 | 1.00 |  |  | 117,937 | 566 | 1.02 (1.00; 1.04) | 0.051 | 1.00 |  |  |
| Gallbladder | <176m, <162w | 100,379 | 19 | 0.98 (0.89; 1.08) | 0.695 | 1.00 | 0.798 | 1.00 | 104,552 | 22 | 1.05 (0.95; 1.17) | 0.344 | 1.00 | 0.838 | 1.00 |
|  | ≥176m, ≥162w | 91,903 | 4 | 1.01 (0.80; 1.28) | 0.922 | 1.00 |  |  | 118,076 | 22 | 1.04 (0.94; 1.15) | 0.442 | 1.00 |  |  |
| Kidney | <176m, <162w | 100,338 | 235 | 1.00 (0.98; 1.04) | 0.746 | 1.00 | 0.210 | 1.00 | 104,534 | 121 | 1.01 (0.96; 1.05) | 0.762 | 1.00 | 0.600 | 1.00 |
|  | ≥176m, ≥162w | 91,858 | 201 | 0.98 (0.94; 1.01) | 0.228 | 1.00 |  |  | 118,049 | 113 | 0.99 (0.95; 1.04) | 0.679 | 1.00 |  |  |
| Leukaemia | <176m, <162w | 100,351 | 173 | **1.04 (1.01; 1.08)** | **0.018** | 0.612 | 0.613 | 1.00 | 104,539 | 83 | 1.03 (0.97; 1.08) | 0.342 | 1.00 | 0.854 | 1.00 |
|  | ≥176m, ≥162w | 91,876 | 177 | 1.03 (0.99; 1.06) | 0.139 | 1.00 |  |  | 118,061 | 117 | 1.01 (0.97; 1.06) | 0.554 | 1.00 |  |  |
| Liver | <176m, <162w | 100,355 | 136 | 0.97 (0.94; 1.00) | 0.091 | 1.00 | 0.078 | 1.00 | 104,542 | 79 | 0.99 (0.94; 1.05) | 0.784 | 1.00 | **0.030** | **0.660** |
|  | ≥176m, ≥162w | 91,898 | 84 | 1.02 (0.98; 1.08) | 0.340 | 1.00 |  |  | 118,068 | 77 | **1.08 (1.03; 1.13)** | **0.002** | 0.09 |  |  |
| Lung | <176m, <162w | 100,259 | 599 | **1.03 (1.01; 1.05)** | **0.003** | 0.114 | 0.146 | 1.00 | 104,469 | 475 | **1.02 (1.00; 1.05)** | **0.030** | 1.00 | **0.019** | **0.437** |
|  | ≥176m, ≥162w | 91,817 | 427 | 1.01 (0.98; 1.03) | 0.601 | 1.00 |  |  | 118,009 | 406 | 0.98 (0.96; 1.01) | 0.173 | 1.00 |  |  |
| Lymphatic | <176m, <162w | 100,261 | 571 | **1.02 (1.00; 1.04)** | **0.045** | 1.00 | 0.100 | 1.00 | 104,480 | 367 | **1.03 (1.00; 1.06)** | **0.024** | 1.00 | 0.273 | 1.00 |
|  | ≥176m, ≥162w | 91,791 | 521 | **1.04 (1.02; 1.06)** | **<0.001** | **<0.001** |  |  | 117,987 | 468 | 1.01 (0.99; 1.03) | 0.525 | 1.00 |  |  |
| Melanoma | <176m, <162w | 100,329 | 249 | 1.02 (0.99; 1.05) | 0.140 | 1.00 | 0.648 | 1.00 | 104,490 | 220 | 1.02 (0.99; 1.06) | 0.165 | 1.00 | 0.550 | 1.00 |
|  | ≥176m, ≥162w | 91,842 | 307 | 1.01 (0.99; 1.04) | 0.295 | 1.00 |  |  | 118,002 | 323 | 1.01 (0.99; 1.04) | 0.350 | 1.00 |  |  |
| Multiple Myeloma | <176m, <162w | 100,359 | 126 | 1.00 (0.96; 1.04) | 0.858 | 1.00 | 0.177 | 1.00 | 104,543 | 89 | 1.02 (0.97; 1.07) | 0.404 | 1.00 | 0.859 | 1.00 |
|  | ≥176m, ≥162w | 91,882 | 102 | 1.04 (0.99; 1.08) | 0.089 | 1.00 |  |  | 118,059 | 99 | 1.01 (0.97; 1.06) | 0.562 | 1.00 |  |  |
| Non-Hodgkin | <176m, <162w | 100,319 | 284 | 1.03 (1.00; 1.05) | 0.074 | 1.00 | 0.181 | 1.00 | 104,510 | 187 | 1.04 (1.00; 1.07) | 0.055 | 1.00 | 0.183 | 1.00 |
|  | ≥176m, ≥162w | 91,846 | 240 | **1.05 (1.02; 1.08)** | **<0.001** | **<0.001** |  |  | 118,025 | 251 | 1.00 (0.97; 1.03) | 0.997 | 1.00 |  |  |
| Oesophagus | <176m, <162w | 100,317 | 235 | **0.96 (0.93; 0.99)** | **0.005** | 0.180 | 0.647 | 1.00 | 104,541 | 67 | 1.03 (0.97; 1.09) | 0.314 | 1.00 | 0.128 | 1.00 |
|  | ≥176m, ≥162w | 91,873 | 173 | **0.96 (0.92; 1.00)** | **0.028** | 0.924 |  |  | 118,061 | 68 | 0.96 (0.91; 1.03) | 0.256 | 1.00 |  |  |
| Oral | <176m, <162w | 100,339 | 173 | 1.01 (0.97; 1.04) | 0.761 | 1.00 | 0.657 | 1.00 | 104,542 | 89 | 0.96 (0.92; 1.01) | 0.140 | 1.00 | 0.061 | 1.00 |
|  | ≥176m, ≥162w | 91,876 | 138 | 1.00 (0.96; 1.04) | 0.823 | 1.00 |  |  | 118,055 | 75 | 1.02 (0.97; 1.07) | 0.431 | 1.00 |  |  |
| Pancreas | <176m, <162w | 100,352 | 197 | 1.01 (0.98; 1.04) | 0.588 | 1.00 | 0.597 | 1.00 | 104,526 | 156 | 1.01 (0.98; 1.05) | 0.471 | 1.00 | 0.454 | 1.00 |
|  | ≥176m, ≥162w | 91,877 | 167 | 1.03 (0.99; 1.06) | 0.162 | 1.00 |  |  | 118,057 | 143 | 1.03 (0.99; 1.07) | 0.146 | 1.00 |  |  |
| Stomach | <176m, <162w | 100,339 | 174 | 0.97 (0.94; 1.00) | 0.056 | 1.00 | 0.784 | 1.00 | 104,542 | 57 | 0.98 (0.92; 1.04) | 0.410 | 1.00 | 0.260 | 1.00 |
|  | ≥176m, ≥162w | 91,877 | 140 | 0.98 (0.94; 1.02) | 0.386 | 1.00 |  |  | 118,069 | 63 | 1.03 (0.98; 1.09) | 0.272 | 1.00 |  |  |
| Thyroid | <176m, <162w | 100,373 | 18 | 1.00 (0.90; 1.12) | 0.928 | 1.00 | 0.644 | 1.00 | 104,536 | 43 | 0.97 (0.91; 1.04) | 0.387 | 1.00 | 0.223 | 1.00 |
|  | ≥176m, ≥162w | 91,903 | 25 | 0.96 (0.87; 1.06) | 0.402 | 1.00 |  |  | 118,062 | 70 | 1.02 (0.97; 1.08) | 0.470 | 1.00 |  |  |
| Prostate /Breast | <176m, <162w | 99,976 | 2,014 | 1.00 (0.99; 1.02) | 0.343 | 1.00 | 0.858 | 1.00 | 104,015 | 1,884 | **1.02 (1.00; 1.03)** | **0.008** | 0.352 | 0.327 | 1.00 |
| Breast | ≥176m, ≥162w | 91,553 | 1,552 | 1.01 (0.99; 1.02) | 0.293 | 1.00 |  |  | 117,435 | 2,399 | 1.01 (1.00; 1.02) | 0.070 | 1.00 |  |  |
| Testis/Cervix | <176m, <162w | 100,378 | 19 | 1.08 (0.96; 1.22) | 0.191 | 1.00 | 0.077 | 1.00 | 104,544 | 26 | 1.03 (0.93; 1.13) | 0.584 | 1.00 | 0.120 | 1.00 |
|  | ≥176m, ≥162w | 91,897 | 37 | 0.96 (0.88; 1.04) | 0.292 | 1.00 |  |  | 118,069 | 24 | 0.91 (0.81; 1.01) | 0.089 | 1.00 |  |  |
| Endometrium | <176m, <162w | -- | -- | -- | -- |  | -- |  | 104,484 | 354 | 1.00 (0.98; 1.03) | 0.918 | 1.00 | 0.718 | 1.00 |
|  | ≥176m, ≥162w | -- | -- | -- | -- |  | -- |  | 118,007 | 292 | 1.00 (0.97; 1.03) | 0.926 | 1.00 |  |  |
| Ovary | <176m, <162w | -- | -- | -- | -- |  | -- |  | 104,500 | 251 | 1.01 (0.98; 1.04) | 0.395 | 1.00 | 0.710 | 1.00 |
|  | ≥176m, ≥162w | -- | -- | -- | -- |  | -- |  | 118,002 | 264 | 1.00 (0.97; 1.03) | 0.943 | 1.00 |  |  |
| Uterine | <176m, <162w | -- | -- | -- | -- |  | -- |  | 104,457 | 380 | 1.00 (0.97; 1.02) | 0.845 | 1.00 | 0.796 | 1.00 |
|  | ≥176m, ≥162w | -- | -- | -- | -- |  | -- |  | 117,991 | 333 | 0.99 (0.97; 1.02) | 0.679 | 1.00 |  |  |

Data is presented as hazard ratio and their 95% intervals in women. Model: Adjusted for age, sex ethnicity, deprivation index, comorbidity, smoking, alcohol consumption, fruit & vegetable, processed meat intake, oily fish, sleep, physical activity, sedentary behaviours and waist circumference. HR: hazard ratio. No data available (--). Padj: P-value adjusted for multiple testing

**Supplementary Table S8:** Association between height and cancer incidence and mortality by IGF-1 concentration levels in men

|  |  | **INCIDENCE** | | |  | **MORTALITY** | | |  |
| --- | --- | --- | --- | --- | --- | --- | --- | --- | --- |
| **Site cancer** | **IGF-1 level** | **Total/event** | **HR 95% CI** | **P value** | P_adjusted_ | **Total/Death** | **HR 95% CI** | **P value** | P_adjusted_ |
| All-cause | Low | 94,400 /7,364 | 1.01 (1.00; 1.01) | **<0.001** | **<0.001** | 96,001 /1,887 | 1.01 (1.00; 1.01) | 0.132 | 1.00 |
|  | High | 94,294 /7,475 | 1.01 (1.00; 1.01) | **<0.001** | **<0.001** | 95,880 /2,099 | 1.01 (1.00; 1.01) | 0.051 | 1.00 |
| Bladder | Low | 96,093 /410 | 1.01 (0.99; 1.02) | 0.303 | 1.00 | 96,198 /74 | 1.00 (0.96; 1.03) | 0.939 | 1.00 |
|  | High | 95,963 /438 | 1.00 (0.99; 1.01) | 0.949 | 1.00 | 96,086 /65 | 1.02 (0.98; 1.05) | 0.387 | 1.00 |
| Brain | Low | 96,161 /144 | 1.02 (0.99; 1.05) | 0.127 | 1.00 | 96,180 /125 | 1.02 (0.99; 1.05) | 0.162 | 1.00 |
|  | High | 96,061 /116 | 1.00 (0.97; 1.03) | 0.961 | 1.00 | 96,074 /106 | 0.99 (0.96; 1.02) | 0.531 | 1.00 |
| Colorectal | Low | 95,979 /746 | 1.01 (0.99; 1.02) | 0.339 | 1.00 | 96,182 /214 | 0.99 (0.97; 1.01) | 0.520 | 1.00 |
|  | High | 95,888 /757 | 0.99 (0.98; 1.00) | 0.203 | 1.00 | 96,072 /203 | 1.01 (0.99; 1.03) | 0.238 | 1.00 |
| Gallbladder | Low | 96,196 /8 | 0.92 (0.82; 1.02) | 0.108 | 1.00 | 96,197 /5 | 0.96 (0.84; 1.10) | 0.525 | 1.00 |
|  | High | 96,086 /15 | 0.96 (0.89; 1.04) | 0.284 | 1.00 | 96,087 /9 | 0.90 (0.82; 0.99) | 0.035 | 1.00 |
| Kidney | Low | 96,147 /229 | 1.01 (0.99; 1.03) | 0.263 | 1.00 | 96,190 /81 | 1.01 (0.98; 1.05) | 0.431 | 1.00 |
|  | High | 96,049 /207 | 0.99 (0.97; 1.01) | 0.308 | 1.00 | 96,083 /72 | 0.98 (0.95; 1.01) | 0.247 | 1.00 |
| Leukaemia | Low | 96,170 /164 | 1.02 (1.00; 1.04) | 0.085 | 1.00 | 96,193 /74 | 1.00 (0.97; 1.04) | 0.990 | 1.00 |
|  | High | 96,057 /186 | 1.04 (1.02; 1.06) | **<0.001** | **<0.001** | 96,082 /86 | 1.04 (1.01; 1.08) | 0.011 | 0.429 |
| Liver | Low | 96,187 /48 | 0.98 (0.94; 1.02) | 0.284 | 1.00 | 96,192 /34 | 0.97 (0.92; 1.02) | 0.199 | 1.00 |
|  | High | 96,066 /172 | 1.00 (0.97; 1.02) | 0.681 | 1.00 | 96,080 /121 | 1.01 (0.98; 1.04) | 0.519 | 1.00 |
| Lung | Low | 96,100 /419 | 1.01 (1.00; 1.03) | 0.056 | 1.00 | 96,147 /315 | 1.01 (0.99; 1.02) | 0.385 | 1.00 |
|  | High | 95,976 /607 | 1.02 (1.00; 1.03) | 0.006 | 0.204 | 96,047 /475 | 1.02 (1.00; 1.03) | 0.013 | 0.494 |
| Lymphatic | Low | 96,093 /530 | 1.02 (1.00; 1.03) | 0.008 | 0.264 | 96,183 /185 | 1.01 (0.99; 1.03) | 0.522 | 1.00 |
|  | High | 95,959 /562 | 1.03 (1.01; 1.04) | **<0.001** | **<0.001** | 96,067 /195 | 1.03 (1.01; 1.05) | 0.010 | 0.400 |
| Melanoma | Low | 96,141 /304 | 1.02 (1.00; 1.04) | 0.020 | 0.640 | 96,198 /33 | 1.03 (0.98; 1.09) | 0.260 | 1.00 |
|  | High | 96,030 /252 | 1.03 (1.01; 1.05) | **<0.001** | **<0.001** | 96,085 /31 | 1.00 (0.95; 1.05) | 0.978 | 1.00 |
| Multiple Myeloma | Low | 96,172 /117 | 1.01 (0.98; 1.04) | 0.620 | 1.00 | 96,194 /28 | 1.00 (0.94; 1.06) | 0.895 | 1.00 |
|  | High | 96,069 /111 | 1.01 (0.99; 1.04) | 0.308 | 1.00 | 96,088 /26 | 1.06 (1.00; 1.12) | 0.040 | 1.00 |
| Non-Hodgkin | Low | 96,147 /251 | 1.03 (1.01; 1.05) | 0.003 | 0.105 | 96,192 /76 | 1.03 (0.99; 1.06) | 0.104 | 1.00 |
|  | High | 96,018 /273 | 1.02 (1.00; 1.04) | 0.051 | 1.00 | 96,079 /81 | 0.99 (0.96; 1.03) | 0.680 | 1.00 |
| Oesophagus | Low | 96,148 /195 | 0.98 (0.96; 1.00) | 0.049 | 1.00 | 96,187 /131 | 0.99 (0.96; 1.02) | 0.429 | 1.00 |
|  | High | 96,042 /213 | 0.98 (0.96; 1.00) | 0.086 | 1.00 | 96,076 /131 | 0.97 (0.94; 0.99) | 0.014 | 0.518 |
| Oral | Low | 96,161 /121 | 1.01 (0.99; 1.04) | 0.317 | 1.00 | 96,197 /28 | 1.02 (0.96; 1.08) | 0.557 | 1.00 |
|  | High | 96,054 /190 | 0.99 (0.97; 1.02) | 0.632 | 1.00 | 96,088 /45 | 1.01 (0.97; 1.06) | 0.557 | 1.00 |
| Pancreas | Low | 96,169 /197 | 1.01 (0.99; 1.03) | 0.248 | 1.00 | 96,176 /168 | 1.02 (0.99; 1.04) | 0.171 | 1.00 |
|  | High | 96,060 /167 | 1.01 (0.99; 1.03) | 0.340 | 1.00 | 96,070 /138 | 1.00 (0.98; 1.03) | 0.924 | 1.00 |
| Prostate | Low | 95,798 /1,896 | 1.00 (0.99; 1.00) | 0.572 | 1.00 | 96,188 /165 | 1.00 (0.97; 1.02) | 0.851 | 1.00 |
|  | High | 95,731 /1,670 | 1.01 (1.00; 1.01) | 0.052 | 1.00 | 96,085 /177 | 1.02 (1.00; 1.04) | 0.087 | 1.00 |
| Stomach | Low | 96,164 /144 | 0.97 (0.95; 1.00) | 0.049 | 1.00 | 96,189 /65 | 1.01 (0.97; 1.04) | 0.781 | 1.00 |
|  | High | 96,052 /170 | 1.00 (0.98; 1.03) | 0.724 | 1.00 | 96,081 /66 | 1.01 (0.98; 1.05) | 0.511 | 1.00 |
| Testis | Low | 96,191 /27 | 1.04 (0.98; 1.10) | 0.204 | 1.00 | 0,001 /1 | 1.00 (1.00; 1.00) | 1.000 | 1.00 |
|  | High | 96,084 /29 | 1.03 (0.98; 1.09) | 0.238 | 1.00 | 0,001 /1 | 1.00 (1.00; 1.00) | 1.000 | 1.00 |
| Thyroid | Low | 96,190 /28 | 1.02 (0.97; 1.08) | 0.440 | 1.00 | 96,198 /2 | 0.96 (0.77; 1.20) | 0.733 | 1.00 |
|  | High | 96,086 /15 | 0.99 (0.91; 1.06) | 0.716 | 1.00 | 96,090 /3 | 0.97 (0.82; 1.15) | 0.741 | 1.00 |

Data is presented as hazard ratio and their 95% intervals in men and women

Model: Adjusted for age, sex ethnicity, deprivation index, comorbidity, smoking, alcohol consumption, fruit & vegetable, processed meat intake, oily fish, sleep, physical activity, sedentary behaviours and waist circumference.IGF-1: Insulin-like growth factor. HR: Hazard Ratio, CI: confidence interval. IGF-1: Low (Mean: 15.7, SD: 2.9), Middle (Mean: 21.1, SD: 1.9), High (Mean: 27.3, SD: 4.2). Padj: P-value adjusted for multiple testing

**Supplementary Table S9:** Association between height and cancer incidence and mortality by IGF-1 concentration levels in women.

|  |  | **INCIDENCE** | | | | **MORTALITY** | | | |
| --- | --- | --- | --- | --- | --- | --- | --- | --- | --- |
| **Site cancer** | **IGF-1 level** | **Total/event** | **HR 95% CI** | **P value** | P_adjusted_ | **Total/Death** | **HR 95% CI** | **P value** | P_adjusted_ |
| All-cause | Low | 94,563 /6,422 | 1.01 (1.01; 1.02) | **<0.001** | **<0.001** | 96,109 /1,352 | 1.01 (1.00; 1.02) | 0.021 | 0.945 |
|  | High | 108,875 /6,819 | 1.01 (1.01; 1.01) | **<0.001** | **<0.001** | 110,518 /1,551 | 1.01 (1.00; 1.02) | 0.035 | 1.00 |
| Bladder | Low | 96,206 /131 | 1.01 (0.98; 1.04) | 0.632 | 1.00 | 96,237 /15 | 1.03 (0.95; 1.11) | 0.529 | 1.00 |
|  | High | 110,590 /140 | 0.99 (0.97; 1.02) | 0.636 | 1.00 | 110,628 /19 | 0.99 (0.92; 1.07) | 0.852 | 1.00 |
| Brain | Low | 96,211 /91 | 1.02 (0.99; 1.06) | 0.176 | 1.00 | 96,227 /79 | 1.01 (0.98; 1.05) | 0.515 | 1.00 |
|  | High | 110,619 /74 | 0.97 (0.93; 1.00) | 0.077 | 1.00 | 110,626 /62 | 0.98 (0.94; 1.02) | 0.358 | 1.00 |
| Breast | Low | 95,682 /2,034 | 1.01 (1.01; 1.02) | **<0.001** | **<0.001** | 96,232 /129 | 1.02 (1.00; 1.05) | 0.084 | 1.00 |
|  | High | 110,074 /1,957 | 1.01 (1.01; 1.02) | **<0.001** | **<0.001** | 110,625 /130 | 1.01 (0.98; 1.04) | 0.553 | 1.00 |
| Cervix | Low | 96,229 /18 | 0.98 (0.91; 1.06) | 0.676 | 1.00 | 96,232 /0 | 1.00 (1.00; 1.00) | 1.000 | 1.00 |
|  | High | 110,621 /30 | 0.98 (0.92; 1.04) | 0.434 | 1.00 | 110,630 /5 | 0.95 (0.83; 1.10) | 0.501 | 1.00 |
| Colorectal | Low | 96,105 /554 | 1.00 (0.98; 1.01) | 0.681 | 1.00 | 96,229 /142 | 1.02 (0.99; 1.05) | 0.111 | 1.00 |
|  | High | 110,501 /540 | 1.01 (1.00; 1.03) | 0.057 | 1.00 | 110,623 /139 | 1.00 (0.97; 1.03) | 0.999 | 1.00 |
| Endometrium | Low | 96,173 /281 | 1.00 (0.98; 1.02) | 0.849 | 1.00 | 96,237 /33 | 1.02 (0.96; 1.08) | 0.486 | 1.00 |
|  | High | 110,552 /344 | 0.98 (0.96; 0.99) | 0.009 | 0.342 | 110,630 /36 | 0.98 (0.93; 1.04) | 0.546 | 1.00 |
| Gallbladder | Low | 96,236 /16 | 1.02 (0.94; 1.10) | 0.635 | 1.00 | 96,237 /9 | 0.99 (0.89; 1.10) | 0.854 | 1.00 |
|  | High | 110,626 /28 | 1.03 (0.97; 1.09) | 0.340 | 1.00 | 110,631 /15 | 1.04 (0.96; 1.13) | 0.366 | 1.00 |
| Kidney | Low | 96,218 /97 | 1.00 (0.97; 1.03) | 0.881 | 1.00 | 96,235 /30 | 0.96 (0.91; 1.02) | 0.179 | 1.00 |
|  | High | 110,599 /133 | 1.00 (0.97; 1.02) | 0.779 | 1.00 | 110,626 /21 | 1.01 (0.94; 1.08) | 0.804 | 1.00 |
| Leukaemia | Low | 96,219 /98 | 1.04 (1.01; 1.08) | 0.013 | 0.481 | 96,231 /36 | 1.06 (1.00; 1.11) | 0.047 | 1.00 |
|  | High | 110,615 /96 | 1.03 (0.99; 1.06) | 0.124 | 1.00 | 110,626 /40 | 1.01 (0.96; 1.06) | 0.634 | 1.00 |
| Liver | Low | 96,229 /56 | 1.01 (0.97; 1.05) | 0.722 | 1.00 | 96,233 /38 | 1.02 (0.97; 1.08) | 0.402 | 1.00 |
|  | High | 110,616 /96 | 1.03 (1.00; 1.07) | 0.051 | 1.00 | 110,623 /69 | 1.04 (1.00; 1.08) | 0.055 | 1.00 |
| Lung | Low | 96,165 /415 | 1.01 (0.99; 1.02) | 0.355 | 1.00 | 96,202 /271 | 1.01 (0.99; 1.03) | 0.467 | 1.00 |
|  | High | 110,548 /454 | 1.00 (0.98; 1.01) | 0.973 | 1.00 | 110,603 /316 | 1.01 (0.99; 1.02) | 0.582 | 1.00 |
| Lymphatic | Low | 96,154 /379 | 1.03 (1.02; 1.05) | <0.001 | <0.001 | 96,225 /105 | 1.04 (1.00; 1.07) | 0.029 | 1.00 |
|  | High | 110,552 /432 | 1.02 (1.01; 1.04) | 0.005 | 0.195 | 110,621 /124 | 1.00 (0.98; 1.03) | 0.790 | 1.00 |
| Melanoma | Low | 96,171 /249 | 1.03 (1.01; 1.05) | 0.004 | 0.160 | 96,237 /15 | 0.96 (0.89; 1.05) | 0.375 | 1.00 |
|  | High | 110,566 /260 | 1.02 (1.00; 1.04) | 0.039 | 1.00 | 110,631 /20 | 0.96 (0.90; 1.04) | 0.310 | 1.00 |
| Multiple Myeloma | Low | 96,220 /92 | 1.02 (0.99; 1.06) | 0.168 | 1.00 | 96,236 /26 | 1.00 (0.94; 1.06) | 0.918 | 1.00 |
|  | High | 110,617 /88 | 1.02 (0.98; 1.05) | 0.334 | 1.00 | 110,629 /24 | 1.04 (0.98; 1.11) | 0.211 | 1.00 |
| Non-Hodgkin | Low | 96,191 /188 | 1.04 (1.01; 1.06) | 0.002 | 0.082 | 96,235 /42 | 1.06 (1.01; 1.11) | 0.029 | 1.00 |
|  | High | 110,581 /239 | 1.02 (1.00; 1.05) | 0.022 | 0.748 | 110,628 /55 | 0.98 (0.94; 1.02) | 0.278 | 1.00 |
| Oesophagus | Low | 96,222 /64 | 0.99 (0.95; 1.03) | 0.522 | 1.00 | 96,235 /38 | 0.99 (0.94; 1.04) | 0.752 | 1.00 |
|  | High | 110,616 /68 | 1.04 (1.00; 1.08) | 0.057 | 1.00 | 110,629 /38 | 1.03 (0.98; 1.09) | 0.236 | 1.00 |
| Oral | Low | 96,221 /67 | 0.98 (0.95; 1.02) | 0.448 | 1.00 | 96,237 /7 | 1.03 (0.91; 1.16) | 0.686 | 1.00 |
|  | High | 110,613 /92 | 0.98 (0.95; 1.02) | 0.329 | 1.00 | 110,631 /20 | 1.00 (0.93; 1.07) | 0.965 | 1.00 |
| Ovary | Low | 96,185 /222 | 1.03 (1.00; 1.05) | 0.018 | 0.630 | 96,231 /103 | 1.02 (0.99; 1.06) | 0.157 | 1.00 |
|  | High | 110,554 /273 | 0.99 (0.97; 1.01) | 0.460 | 1.00 | 110,625 /139 | 1.00 (0.97; 1.03) | 0.886 | 1.00 |
| Pancreas | Low | 96,215 /140 | 0.99 (0.97; 1.02) | 0.600 | 1.00 | 96,225 /123 | 0.99 (0.97; 1.02) | 0.733 | 1.00 |
|  | High | 110,603 /154 | 1.03 (1.00; 1.06) | 0.031 | 1.00 | 110,619 /129 | 1.02 (0.99; 1.04) | 0.291 | 1.00 |
| Stomach | Low | 96,224 /55 | 0.99 (0.95; 1.04) | 0.707 | 1.00 | 96,234 /27 | 1.02 (0.96; 1.08) | 0.554 | 1.00 |
|  | High | 110,622 /62 | 1.03 (0.99; 1.07) | 0.177 | 1.00 | 110,628 /37 | 1.01 (0.96; 1.06) | 0.735 | 1.00 |
| Thyroid | Low | 96,215 /52 | 1.02 (0.98; 1.07) | 0.276 | 1.00 | 96,236 /0 | NA. |  |  |
|  | High | 110,617 /52 | 1.03 (0.99; 1.08) | 0.172 | 1.00 | 110,630 /3 | 0.87 (0.72; 1.06) | 0.165 | 1.00 |
| Uterine | Low | 96,156 /302 | 1.00 (0.98; 1.02) | 0.919 | 1.00 | 96,237 /51 | 1.03 (0.98; 1.08) | 0.208 | 1.00 |
|  | High | 110,529 /388 | 0.98 (0.96; 1.00) | 0.014 | 0.504 | 110,628 /52 | 0.98 (0.94; 1.03) | 0.461 | 1.00 |

Data is presented as hazard ratio and their 95% intervals in men and women

Model: Adjusted for age, sex ethnicity, deprivation index, comorbidity, smoking, alcohol consumption, fruit & vegetable, processed meat intake, oily fish, sleep, physical activity, sedentary behaviours and waist circumference.IGF-1: Insulin-like growth factor. HR: Hazard Ratio, CI: confidence interval. IGF-1: Low (Mean: 15.7, SD: 2.9), Middle (Mean: 21.1, SD: 1.9), High (Mean: 27.3, SD: 4.2). Padj: P-value adjusted for multiple testing

**
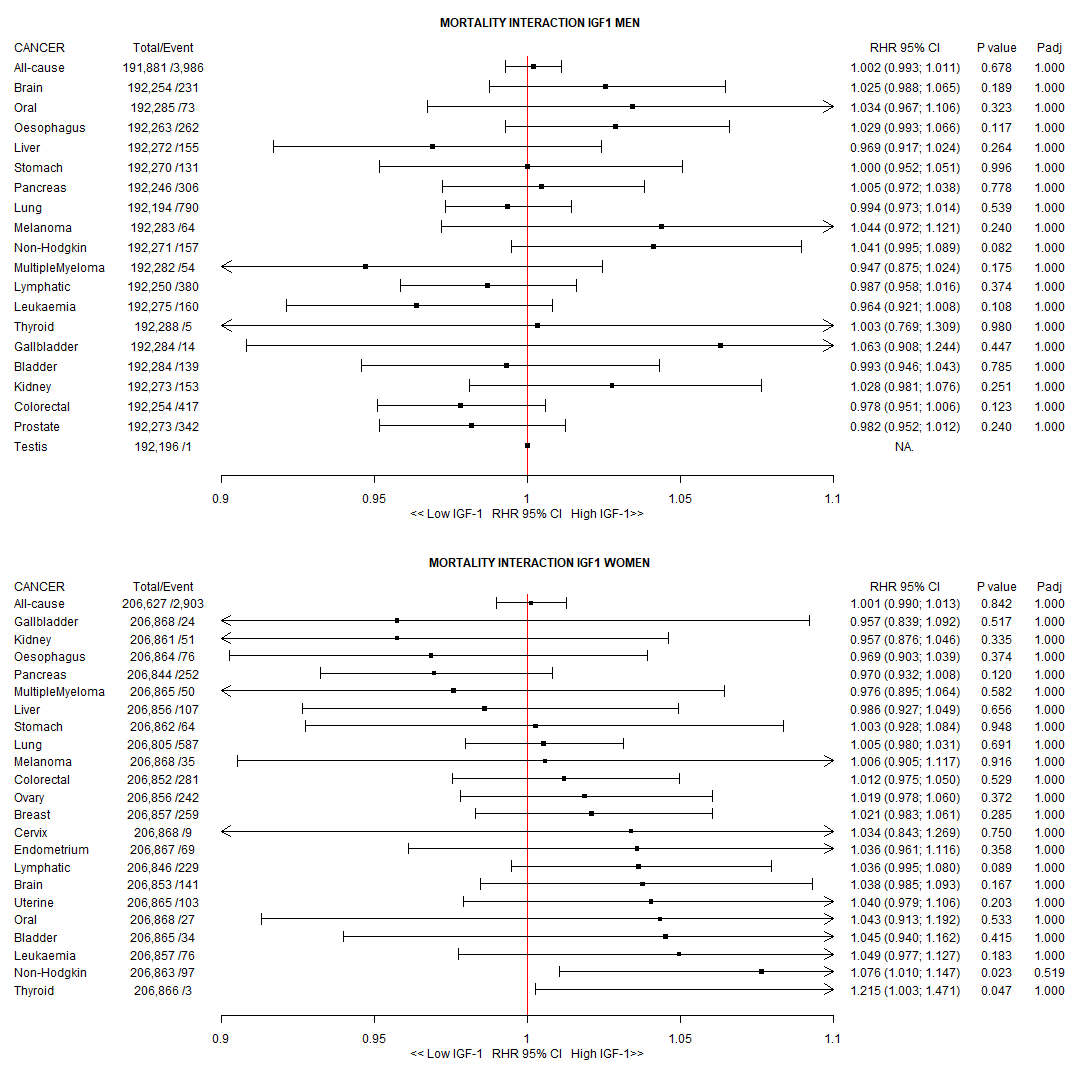
**

**Supplementary Figure S4.** Ratio of HR of low to high IGF-1 level for the association of height with mortality of 24 cancer sites men and women.

Data presented as ratio of hazard ratio (interaction term of IGF-1 level and height) and their 95% CI per 5-cm increment in height. Models were adjusted for age, ethnicity, deprivation index, comorbidity, smoking, alcohol consumption, fruit & vegetable, processed meat intake, oily fish, sleep, physical activity, sedentary behaviours and waist circumference. Padj: P-value adjusted for multiple testing
